# Respiratory Failure and Death in Vulnerable Premature Children With Lower Respiratory Tract Illness

**DOI:** 10.1093/infdis/jiaa046

**Published:** 2020-02-01

**Authors:** Gaston Ofman, Brad Pradarelli, Mauricio T Caballero, Alejandra Bianchi, Luciano Alva Grimaldi, Andrea Sancilio, Karina Duenas, Andrea Rodriguez, Fernando Ferrero, Adrian Ferretti, Silvina Coviello, Fausto M Ferolla, Patricio L Acosta, Eduardo Bergel, Romina Libster, Fernando P Polack

**Affiliations:** 1 Fundación INFANT, Buenos Aires, Argentina; 2 Consejo Nacional de Investigaciones Científicas y Técnicas (CONICET), Buenos Aires, Argentina; 3 Hospital Zonal General de Agudos “Lucio Melendez”, Buenos Aires, Argentina; 4 Hospital Interzonal General de Agudos “Evita” de Lanus, Buenos Aires, Argentina; 5 Hospital Zonal General de Agudos Descentralizado “Evita Pueblo” de Berazategui, Buenos Aires, Argentina; 6 Hospital General de Niños “Pedro de Elizalde”, Buenos Aires, Argentina

**Keywords:** prematurity, respiratory failure, respiratory infection

## Abstract

**Background:**

Efforts to better understand the risk factors associated with respiratory failure (RF) and fatal lower respiratory tract infection (LRTI) in premature children in developing countries are necessary to elaborate evidenced-based preventive interventions. We aim to characterize the burden of respiratory syncytial virus (RSV) and human metapneumovirus (hMPV) LRTI in premature children and determine risk factors for RF and fatal illness in a vulnerable population.

**Methods:**

This is a prospective, population-based, cross-sectional study. Subjects with severe LRTI were enrolled during respiratory season. Risk factors for RF and death in premature infants were investigated.

**Results:**

A total of 664 premature children participated. Infant’s hospitalization rate due to LRTI was 82.6/1000 (95% confidence interval [CI], 68.6–96.7/1000). Infant’s RSV and hMPV rates were 40.9/1000 (95% CI, 36.3–45.6/1000) and 6.6/1000 (95% CI, 3.9–9.2/1000), respectively. The RF rate was 8.2/1000 (95% CI, 4.9–11.5/1000). The LRTI mortality was 2.2/1000 (95% CI, 0.7–3.7/1000); for RSV, the rate was 0.8/1000 (95% CI, 0–1.7/1000) with a case-fatality ratio of 1.8%. Never breastfeeding, malnutrition, younger than 6 months, congenital heart disease, and lower hematocrit were risk factors for RF. Experiencing pneumonia, pneumothorax, sepsis, or apnea were clinical determinants of poor outcomes.

**Conclusions:**

Premature children under 2 years old in vulnerable environments experience RF and death more often than term counterparts. Modifiable risk factors associated with poor outcomes should prompt evidence-based interventions.

## Teaser

This prospective, population-based, cross-sectional studied factors associated with respiratory failure and fatal LRTI in premature infants in developing countries. Six hundred sixty-four premature children participated, 60 developed respiratory failure, and 15 died. Several risk factors were identified for future interventions.

**(See the Editorial Commentary by Nicholson and Piedra, on pages 1070–2.)**

Children born before 37 weeks of gestation are more likely to die than their full-term counterparts. Mortality rates increase proportionately with decreasing gestational age and birth weight [1]. Approximately one third of all infant mortality in the United States is associated with prematurity, which also remains a leading cause of death in children under 5 years of age worldwide [2, 3]. In addition to the direct effects of premature birth on infant health, lower respiratory tract infections (LRTIs) are the most frequent cause of hospitalization in the world, and they impose a greater burden on premature children [4]. It is estimated that LRTI due to respiratory syncytial virus (RSV), a leading cause of acute lung disease, account for 63.8 hospitalizations and 1.04 deaths per 1000 premature children every year worldwide [5]. The vast majority of these deaths occur in developing countries, but the characterization of risk factors for near-fatal and fatal outcomes in premature infants and young children in low-income regions is limited [6]. The contribution of pathogens other than RSV, such as human metapneumovirus (hMPV), to these outcomes is also unclear.

Efforts to better understand the socioeconomic, biological, and clinical risk factors associated with disease leading to respiratory failure (RF) and fatal LRTI in premature children in developing countries are necessary to elaborate evidenced-based preventive interventions. Living environments may contribute to adverse events during gestation, the neonatal period, and infancy, and impact on the severity of LRTI. Biological characteristics associated with prematurity are known to enhance the risk of poor outcomes [7–9]. Clinical management of vulnerable children in facilities with limited resources may also compromise results [10–12]. Defining and weighing these risk factors through large prospective studies with research capacity to collect data in low-income populations is important.

We conducted a prospective study to investigate the burden of severe and fatal viral LRTI during 3 consecutive years in a catchment population of 56 560 children under age 2 years in a vulnerable region of Argentina. In this population, we defined rates of RF and fatal disease for premature children with RSV and/or hMPV, and we examined risk factors for RF to identify future interventions that can mitigate this serious problem.

## METHODS

### Study Population

Between 2011 and 2013, we conducted a prospective, population-based, cross-sectional, multicenter study to determine the burden and risk factors for near-death episodes (RF) and mortality due to LRTI in a vulnerable region of Argentina with a catchment population of 56 560 children under age 2 per year [13]. Rates were estimated using information from population statistics of the Buenos Aires region and the neighbor city of La Plata. The region is home to an estimated 361 000 children under 2 years of age. Approximately, 170 000 of these subjects have no medical insurance [14]. Details of the program are provided elsewhere [13, 15].

Eligible subjects for our study were children under 2 years of age, born before 37 weeks of gestation, and rehospitalized due to severe LRTI. Severe LRTI was defined as the sudden onset of cough, tachypnea, wheezing, retractions, and/or crackles with or without fever, and either an oxygen saturation <93% at rest when breathing room air, or arriving to the emergency room (ER) receiving oxygen supplementation due to acute respiratory symptoms [15]. Respiratory failure was defined as an episode (1) requiring mechanical ventilation (excluding noninvasive ventilation or continue positive airway pressure devices) or (2) resulting in death during hospitalization due to LRTI. Hospitalization and clinical management decisions were prerogatives of the individual ER physicians. The institutional review boards at each participating hospital approved the study. Parents and/or guardians of candidates were invited to participate by study pediatricians when their children met inclusion criteria. Informed consent was obtained from all participating parents or guardians.

### Study Variables

Information on socioeconomic and biological risk factors was collected prospectively from all participants, using questionnaires. Follow-up questionnaires were used daily to collect data on clinical course until discharge or death (Supplemental Material). For fatal cases, we reviewed medical records to verify and/or obtain specific information. Lower hematocrit was defined as having a hematocrit under the mean for the population (30.6%). Underweight was defined as a weight Z-score upon admission ˂2 standard deviation. Pediatricians in Argentina follow the diagnostic consensus elaborated by the Argentinean Society of Pediatrics [16]. Pneumonia is defined as an acute infection of pulmonary parenchyma with clinical signs of alveolar occupation ±radiologic findings of opacity; bronchiolitis is defined as an episode of respiratory distress associated with wheezing in children less than 2 years of age in the setting of a viral illness. The Argentinean Society of Thoracic Surgery defines pneumothorax as the presence of air in the pleural space. Clinical significance was attributed when the pneumothorax affected respiratory and/or hemodynamic stability [17].

### Virologic Studies

Nasopharyngeal (NP) secretions were obtained from children with severe LRTI by pediatricians during the respiratory season and tested for RSV and hMPV by real-time, reverse-transcriptase polymerase chain reaction (RT-PCR), as previously described [13, 15]. The respiratory season started every year on detection of 2 cases of severe RSV LRTI at 1 of the 12 participating institutions through the hospital’s surveillance system. The season ended when no patients were admitted with RSV LRTI to 4 of the 12 participating hospitals during the same week [13, 15]. The duration of 3 seasons were 15 weeks, 17 weeks, and 16 weeks, respectively [18].

### Statistical Analysis

Respiratory failure and death rates in premature children were calculated using data from the State Census on premature children in our region [14]. Children with RSV and hMPV coinfection were assigned to both viral illnesses and consequently considered to estimate both RSV and hMPV rates. Student’s *t* tests and χ ^2^ tests were used to compare risk factors where appropriate. We performed a hierarchical multilevel regression model that incorporated socioeconomic variables (level 1), biologic vulnerabilities (level 2), and clinical complications (level 3). We used a conceptual basis considering the interrelationship between variables with biological and social interpretation. A decision was made to divide each group of independent variables more distal (socioeconomic) and more proximal (biologic and clinical) to the outcome. Thus, factors are organized, directly or indirectly, factors grouped later. Variables with a *P* < .05 were selected from the univariable analysis for the hierarchical model [19].

## RESULTS

### Burden of Severe Lower Respiratory Track Infection in Premature Children

A total of 4405 children were identified with severe LRTI during 3 consecutive respiratory seasons between 2011 and 2013; parents or guardians of 3947 (97.6%) consented to participate in this study [15]. Of these children, 678 (17.1%) were born prematurely and 14 had no recorded clinical evolution; therefore, 664 total preterm children were deemed eligible for analysis. Among these children, 51.6% were infected with RSV and 8.1% were infected with hMPV. Sixty (9%) children experienced RF; 45 survived after receiving mechanical ventilation and 15 (2.3%) died. These 60 children with RF included 28 infected with RSV and 2 with hMPV. Among the 15 fatal cases, 6 had RSV and none had hMPV.

Our study was conducted in a catchment infant population of 28 280 per year [14]. The rate of prematurity in our area was 8% [2]. Therefore, our study was estimated to monitor 2262 premature infants and 4454 children under 2 years each year. The hospitalization rate for live-born premature infants (0–12 months of age) due to LRTI was 82.6/1000 (95% confidence interval [CI], 68.6–96.7/1000) and for live-born premature children (0–24 months) the rate was 48.9/1000 (95% CI, 39.9–57.9/1000) (Table 1). Rates for RSV and hMPV were 40.9/1000 (95% CI, 36.3–45.6/1000) and 6.6/1000 (95% CI, 3.9–9.2/1000), respectively.

**Table 1.** Disease Rates in Premature Infants (0–12 Months) and Children (0–24 Months)^a^

	Infants		Children	
	n	Rates	n	Rates
Hospitalization	561	82.6 (68.6–96.7)	664	48.9 (39.9–57.9)
RSV severe LRTI	278	40.9 (36.3–45.6)	333	24.5 (21.8–27.1)
hMPV severe LRTI	45	6.6 (3.9–9.2)	50	3.6 (1.1–4.8)
All RF	56	8.2 (4.9–11.5)	60	4.4 (2.8–5.9)
RSV RF	26	3.83 (0.6–7)	28	2 (0–3.7)
hMPV RF	2	0.2 (0–0.5)	2	0.1 (0–0.2)
Death	15	2.21 (0.7–3.7)	15	1.1 (0.3–1.8)
RSV death	6	0.8 (0–1.7)	6	0.4 (0–0.8)

Abbreviations: hMPV, human metapneumovirus; LRTI, lower respiratory track infection; RF, respiratory failure; RSV, respiratory syncytial virus.

^a^Number of infants (0–12 months) and children (0–24 months) represents all participants with specific criteria for the duration of the study (3 years). Rates were based on the local census of live-born premature infants per year and represent mean values with 95% confidence intervals per 1000 live-born premature infants or children per year.

Ten cases were coinfections with RSV and hMPV, but none of the RSV and hMPV cases experienced RF nor died. Respiratory syncytial virus and hMPV RF and deaths are detailed in Table 1. The case-fatality ratio (CFR) for RSV was 1.8%.

### Socioeconomic Vulnerability Associated With Respiratory Failure Due to Lower Respiratory Track Infection

Forty-three percent of 664 premature children had an incomplete vaccination schedule, and more than two thirds were born to mothers with no or incomplete high school education (77%). Precarious homes were the rule, with no running water (19%), no sewage system (60%), construction using tin or mud (23%), and crowding (27%) (Table 2).

**Table 2.** General Population Demographics and Overall Frequency of Risk Factors in Preterm vs Term Infants (Described Here for Comparison)^a^

Population Demographics	Preterm	Term
	n/N (%)	n/N (%)
Socioeconomic		
Access to healthcare		
Incomplete vaccination	264/608 (43.4)	1180/2960 (39.8)
At least 1 previous visit in this episode	231/394 (58.6)	1274/2008 (63.4)
Vulnerable Mother		
Adolescent mother	125/615 (20.3)	414/2993 (13.8)*
Late childbearing	66/615 (10.7)	278/2993 (9.2)
No/incomplete high school education	496/641 (77.4)	2410/3093 (77.6)
Precarious Home		
No running water	121/649 (18.6)	846/3131 (27)*
No sewage system	387/641 (60.4)	1945/3084 (63)*
Tin, mud home	151/634 (23.8)	748/3031 (24.6)
Tobacco smoking at home	414/657 (63.0)	1846/3193 (57.8)
Crowding	155/581 (26.7)	812/2783 (29.1)
Biological		
Young and Light		
Extremely preterm	25/664 (3.8)	-
Very Preterm	88/664 (13.2)	-
Moderate/late preterm	551/664 (83.0)	-
Age ≤6 months	400/625 (64.0)	1955/3210 (60.9)
Underweight	166/628 (26.4)	270/3056 (8.8)*
Comorbidities		
Congenital heart disease	47/642 (7.3)	67/3112 (2.1)*
Down syndrome	4/664 (0.6)	16/3269 (0.4)
Vulnerable Lungs		
Mother smoked while pregnant	146/637 (22.9)	612/3120 (19.6)
Male Sex	370/659 (56.1)	1801/3163 (56.9)
Recurrent wheeze	132/664 (19.9)	766/3269 (23.4)
Never breastfed	126/581 (21.7)	399/2990 (13.3)*
Ventilated at birth	189/428 (44.2)	44/3269 (1.3)*
Clinical		
Complications		
Apnea	34/664 (5.1)	33/3269 (1)*
Pneumonia	174/664 (26.2)	814/3269 (24.9)
Sepsis	22/664 (3.3)	37/3269 (1.1)*
Pneumothorax	11/664 (1.8)	17/3269 (0.5)*
Bronchiolitis	464/664 (69.9)	2377/3269 (72.7)
Perinatal infection	10/664 (1.5)	28/3269 (0.8)*
Nasopharyngeal Swabs		
RSV	333/645 (51.6)	1855/3214 (57.7)*
HMPV	50/620 (8.1)	268/3169 (8.4)

Abbreviations: hMPV, human metapneumovirus; RSV, respiratory syncytial virus.

^a^Comparison of variables in preterm and term infants (n) for the total number of infants with available data for each variable (N) and resulting rate. Adolescent mothers were those ≤18 years old. Late childbearing was defined in mothers ≥35 of age. Extreme preterm: born before 28 weeks of gestation. Very preterm: born between 28 and 32 weeks of gestation. Moderate/late preterm: born between 32 and 36 weeks of gestation. Underweight: Z-score ≤2 standard deviation.

*, *P* < .05.

Therefore, we examined the role of poor access to healthcare, vulnerable mothers, and living conditions as determinants of poor outcomes during LRTI. Most risk factors were distributed evenly among children with RF and children admitted with severe LRTI who did not develop RF (Table 3). Only a lack of or incomplete high school education in their mothers affected RF rates (10.2% vs 3.6%).

**Table 3.** Univariable Analysis of Risk Factors for RF in Premature Infants With Severe LRTI^a^

	Premature Infants (n = 664)		
	RF [n/N (%)]	No RF [n/N (%)]	OR (95% CI)
Socioeconomic			
Access to Healthcare			
Incomplete vaccination	20/45 (44.44)	244/563 (43.33)	1.05 (0.57–1.93)
At least a previous visit in this episode	28/46 (60.86)	203/348 (58.33)	1.11 (0.59–2.09)
Vulnerable Mother			
Adolescent mother	11/49 (22.44)	114/566 (20.14)	1.15 (0.57–2.32)
Late childbearing	5/49 (10.2)	61/566 (10.77)	0.94 (0.36–2.46)
No/incomplete high school education	46/51 (90.19)	450/590 (76.27)	**2.86 (1.12–7.34)** ^b^
Living Conditions			
Precarious home	4/51 (7.84)	38/600 (6.33)	1.26 (0.43–3.68)
Tobacco smoking at home	36/53 (67.92)	378/604 (62.58)	1.27 (0.7–2.31)
Crowding	12/46 (26.08)	143/535 (26.72)	0.97 (0.49–1.92)
Biological			
Young and Light			
Extremely preterm	2/60 (3.33)	23/604 (3.8)	0.87 (0.2–3.79)
Very preterm	12/60 (20)	76/604 (12.58)	1.74 (0.88–3.42)
Moderate/late preterm	46/60 (76.66)	505/604 (83.6)	0.64 (0.34–1.22)
Age ≤6 months	44/50 (88)	356/575 (61.91)	**4.51 (1.89–10.76)**
Underweight	28/52 (53.84)	138/576 (23.95)	**3.7 (2.08–6.6)**
Comorbidities			
Congenital heart disease	9/55 (16.36)	38/586 (6.48)	**2.82 (1.28–6.2)**
Vulnerable Lungs			
Mother smoked while pregnant	15/48 (31.25)	131/589 (22.24)	1.59 (0.84–3.02)
Male sex	39/60 (65)	331/599 (55.25)	1.5 (0.86–2.62)
Recurrent wheeze	12/60 (20)	120/604 (19.86)	1.01 (0.52–1.96)
Never breastfed	27/56 (48.21)	152/558 (27.24)	**2.49 (1.43–4.34)**
Ventilated at birth	26/46 (56.52)	163/382 (42.67)	1.75 (0.94–3.24)
Clinical			
Laboratory Tests			
Low hematocrit	34/54 (62.96)	163/382 (42.67)	**1.99 (1.11–3.56)**
Nasopharyngeal Swabs			
RSV	28/37 (75.67)	305/588 (55.27)	0.9 (0.52–1.54)
HMPV	2/51 (3.92)	48/588 (8.43)	0.44 (0.1–1.88)
Complications			
Apnea	12/60 (20)	22/602 (3.65)	**6.61 (3.09–14.18)**
Pneumonia	25/60 (41.66)	149/604 (24.66)	**2.18 (1.26–3.76)**
Sepsis	19/60 (31.66)	3/604 (0.49)	**92.84 (26.38–326.66)**
Pneumothorax	9/60 (15)	3/604 (0.49)	**35.35 (9.28–134.68)**
Bronchiolitis	40/60 (66.66)	424/604 (70.19)	0.85 (0.48–1.49)
Perinatal infection	2/22 (9.09)	8/174 (4.59)	2.08 (0.41–10.46)

Abbreviations: CI, confidence interval; hMPV, human metapneumovirus; LRTI, lower respiratory track infection; OR, odds ratio; RF, respiratory failure; RSV, respiratory syncytial virus.

^a^Precarious home: no running water, no sewage system and/or tin/mud home. “n” represents the number of children with RF or without RF (no RF) and experienced a specific risk factor. “N” represents the total number of children with available data for each variable.

^b^Bold indicates *P* < .05.

### Biological Vulnerability Associated With Respiratory Failure Due to Lower Respiratory Track Infection

Most children were moderate to late preterm (83%); 400 of 664 (60%) experienced severe LRTI in the first 6 months of life. Forty-seven premature children had congenital heart disease; 4 had Down’s Syndrome. Approximately 1 in 4 mothers smoked during pregnancy. Breastfeeding rates were high for premature babies [20], with only 21.7% of children never receiving human milk (Table 2), and higher than term children (13.3%).

To further explore biological risk factors influencing poor outcomes, we investigated whether being “young and light,” having congenital diseases, and/or lung vulnerabilities affected the outcomes (Table 3). More children with RF presented before 6 months of age compared with children with no RF (12% vs 0.3%). In addition, being underweight (Z-score less than 2 standard deviations), never breastfeeding, and/or having congenital heart disease significantly increased the risk of RF.

### Clinical Vulnerability Associated With Respiratory Failure Due to Lower Respiratory Track Infection

The most frequent diagnosis among children with severe LRTI was bronchiolitis (70%); 52% was caused by RSV. Pneumonia was observed in 175 hospitalized children, 49% with RSV. Bacterial pathogens in children with pneumonia were identified in 25 (14%) blood cultures; 10 of these cases simultaneously with detection of RSV in NP secretions. Fourteen cases of bacteremia were due to Gram-negative bacteria (5 *Escherichia coli*, 3 *Klebsiella pneumoniae*, 2 *Pseudomonas aeruginosa*, and 1 each with *Acinetobacterbaumannii*, *Haemophilus* spp, *Moraxella catarrhalis*, and *Proteus mirab*ilis). Eleven blood cultures grew Gram-positive cocci (3 with *Staphylococcus aureus*, 3 with *Streptococcus pneumoniae*, 3 with coagulase-negative staphylococci, and 1 each with *Enterococcus faecalis* and *Streptococcus viridans*). An additional blood culture grew *Candida tropicalis*. Clinically significant pneumothoraxes affected 11 children; 6 of them infected with RSV, and with an incidence 3 times higher than term children. Thirty-four children experienced apnea; RSV was detected in 11 of them. In contrast to term children, and consistent with the history of prematurity, more preterm children were ventilated at birth.

Assessing clinical characteristics potentially affecting the risk for RF revealed no association between RF and RSV or hMPV. Experiencing apnea, a lower hematocrit, pneumonia, sepsis, or a clinically significant pneumothorax increased the risk of having RF (Table 3).

### Hierarchical Analysis of Risk Factors of Respiratory Failure in Premature Children

Finally, we investigated the hierarchical relationship between these environmental, biological, and clinical factors with RF (Table 4). Our analysis continued to show a significant role for lack of and/or partial maternal high school education, age less than 6 months at presentation to the hospital, and being underweight in RF. Moreover, experiencing a clinically significant pneumothorax, sepsis, and apnea also continued to significantly contribute to the risk of having RF, demonstrating that public health interventions at multiple levels can impact the fate of premature children with LRTI.

**Table 4.** Hierarchical Multilevel Analysis of Risk Factors for RF Due to Severe LRTI in Premature Infants^a^

Risk factor	Level 1	Level 2	Level 3
	*P* Value	*P* Value	*P* Value
No/incomplete high school education	**.023**	*.2*	*.107*
Age ≤6 months		**.009**	*.031*
Underweight		**.019**	*.126*
Never breastfed		**.03**	*.558*
Apnea			**<.001**
Pneumonia			.09
Sepsis			**<.001**
Pneumothorax			**.04**

Abbreviations: LRTI, lower respiratory track infection; RF, respiratory failure.

^a^In levels 2 and 3 of the hierarchical analysis, the variables analyzed in the previous level were used only to adjust the next level variables (italicized; gray cells). Bold indicates *P* < .05.

## Discussion

Our study shows that in vulnerable regions, all-cause severe LRTI leading to hospital admission in premature children can be extremely frequent, affecting approximately 10 percent of all children. Risk factors for poor outcomes are diverse, but they are heavily associated with acute clinical events, such as pneumothoraxes and secondary bacterial infections, and—to a lesser extent—with biological vulnerabilities, including cardiac congenital malformations and age at presentation. The RSV hospitalization rate at ~5% was similar to global estimates of 6.3% [4]. Respiratory syncytial virus hospital-based preterm mortality more than tripled that in children born at term (0.8 vs 0.23/1000), and the RSV-specific CFR for preterm babies doubled the CFR in term babies (1.8% vs 0.79%) [15]. It is interesting to note that the impact of hMPV waned with increasing disease severity, representing 1 of 7 RSV admissions, 1 of 20 of RSV RF, and causing no deaths in premature babies during the 3 years of our study.

Approximately half of all children in our study were not up to date in their vaccinations, two thirds of their mothers did not start or complete secondary school, and precarious homes were the rule: crowded homes often built with tin or mud, sometimes with no running water, and frequently with no sewage system dominated the region. Similar socioeconomic conditions were associated with community deaths due to LRTI in the region [39]. However, the socioeconomic background can also affect health outcomes in premature babies of wealthier nations. A recent French study of 589 premature children showed that living in deprived neighborhoods was associated with a higher risk of rehospitalization, 44% of which were a consequence of LRTI [21]. In fact, premature children living with families of low socioeconomic status have higher readmission rates and ER visits [22]. In addition, several reports described a link between maternal education and poor early neonatal outcomes, from low birth weight to infant mortality [23–25].

Most studies of risk factors for severe RSV LRTI in moderate to late preterm children were conducted in industrialized nations [26–32] and investigated variables associated with hospitalization or severe LRTI, not RF or death. Factors affecting the most severe end-of-the spectrum C with LRTI have remained more elusive. Although male sex [28] and being underweight [33, 34] were associated with severe RSV LRTI in preterm children, only the latter was a significant risk factor for RF in our study. It is interesting to note that never breastfeeding—highlighting the critical importance of human milk in infant health [35–37]—and experiencing a severe acute lung illness before the age of 6 months [6, 38] were predictors of poor outcomes. In a global retrospective assessment of risk factors in children, age younger than 6 months and comorbidities enhanced the risk of death due to RSV [15, 39, 40]. In fact, comorbidities dominated risk factors particularly in high-income countries [41]. Congenital heart disease was associated with poor outcomes in this study. It is clear that living conditions—pertinent to the whole region and not necessarily distinct between hospital deaths and survivors—bear a greater influence in the developing world than that in industrialized nations.

The impact of clinical complications, as we previously highlighted in a study of post-neonatal children, remains a strong driver of poor LRTI outcomes also in premature patients [15, 39]. For instance, pneumothorax significantly increases the risk of death and RF in all children with RSV LTRI [15]. In this study, ~90% of premature subjects experiencing a pneumothorax progressed to experience RF. It is conceivable that preterm children, having immature lungs and less ventilatory reserve, can experience an acute deterioration in the presence of a ventilation/perfusion mismatch more readily than older counterparts [42].

Reports informing on the incidence of bacterial pneumonia and sepsis specifically in premature children are scarce and conflicting. In one study, a younger gestational age at birth associated with higher incidence of developing serious bacterial infections (SBIs) [43]. However, a second retrospective report failed to detect a higher incidence of SBI in ex-premature children who had a full or partial septic work-up in the ER compared with term children [44], whereas a third study identified a concurrent bacterial infection in 9.5% of preterm versus 3.1% of term children with RSV LRTI [45]. In the United States, the incidence of bacteremia in children <18 years old requiring hospitalization for pneumonia was 7% (4.7%–10.1%) when a blood culture was obtained [46] but only 2.5% in a second retrospective study excluding children with chronic conditions [47]. We detected bacteremia in 14.2% of our preterm patients; 40% of all cases of bacteremia had RSV in Nasopharynx (51% of all admissions and 49% of pneumonias were infected with RSV). A recent trial immunizing pregnant women to prevent severe RSV LRTI in children through passive transfer of specific antibody showed significant long-term protection against all-cause pneumonia in vaccinated subjects for at least 6 months [48]. However, bacterial pneumonia remains a major cause of death in vulnerable populations [49]. Nevertheless, numerous strategies under evaluation to prevent RSV disease may hence provide extended benefits to children, including, in some cases, premature children, with far-reaching implications beyond the virus itself [48].

Our study has limitations. Among them, we studied hospital-based RF and fatalities but not community deaths. However, a previous study in our community did not identify prematurity as a risk factor for infant mortality [39]. It is interesting to note that neonatal intensive care unit (NICU) admission at birth associated with home death, but it was linked to congenital infections (TORCH) and human immunodeficiency virus. In addition, in-hospital deaths were insufficient to power a hierarchical analysis. In this study, we did not examine the role of other viruses including influenza and human parainfluenza virus type 3 (hPIV3). In an earlier evaluation of another population of extremely premature babies, we observed hPIV3 to exert more severe disease than influenza; in particular, because its seasonality extended beyond the respiratory season [40]. In our study, the virologic evaluation was performed on admission or hospitalization; therefore, we are not able to precisely identify RSV nosocomial infection. Finally, we did not query families retrospectively about receiving a diagnosis of bronchopulmonary dysplasia. However, we did study the association between receiving mechanical ventilation in the NICU and poor outcomes.

Nevertheless, this study also has important strengths. First, to our knowledge, this is the first large prospective study that gathered detailed socioeconomic, biologic, and clinical information from a large population of severely ill premature children from a vulnerable region. Second, we monitored the population for 3 consecutive years allowing robust conclusions. Third, RT-PCR results permitted us to explore rates of disease burden specific to 2 important viral agents, RSV and hMPV, and clarify their pathogenic association with socioeconomic and clinical risk factors. In addition, traditional multivariable analyses treat observations as if they were independent. Using clusters, or groups, we considered the interrelationship between variables with biological, social, and clinical interpretation to better target interventions. Finally, because we previously studied viral illnesses at the hospital and in the community in this region, we were able to contextualize our data in premature children against findings in other studies [6, 15, 50].

## Conclusions

In summary, this prospective, multicenter study shows that premature children living in a vulnerable context are frequently hospitalized due to severe LRTI. Although not a specific risk factor for RF, many children with life-threatening illness are infected with RSV. In contrast, hMPV is more often responsible for milder disease. However, regardless of the infecting virus, many factors contribute to RF. Moreover, some factors, such as maternal education, being deprived of human milk, lower hematocrit, or being underweight, are modifiable. Other factors, including age at presentation or congenital heart disease, remain a challenge that active and/or passive prevention strategies against specific pathogens may resolve. Finally, individual clinical events potentially associated with medical practice, such as significant pneumothoraxes or secondary bacterial infections, highlight the importance of emphasizing medical training and hospital standard procedures as vulnerable regions access life-saving technologies.

## Supplementary Data

Supplementary materials are available at *The Journal of Infectious Diseases* online. Consisting of data provided by the authors to benefit the reader, the posted materials are not copyedited and are the sole responsibility of the authors, so questions or comments should be addressed to the corresponding author.

jiaa046_suppl_Supplementary-Material

## Notes

***Acknowledgments*.** This work was funded by a grant from the Bill & Melinda Gates Foundation (OPP1018614).

***Potential conflicts of interest.*** All authors: No reported conflicts of interest. All authors have submitted the ICMJE Form for Disclosure of Potential Conflicts of Interest.
