# Supplementary material for: Respiratory Failure and Death in Vulnerable Premature Children With Lower Respiratory Tract Illness
Source: J Infect Dis. 2020 Feb 1;222(7):1129–37. doi: 10.1093/infdis/jiaa046 (PMC7459133; doi:10.1093/infdis/jiaa046)
Supplement: jiaa046_suppl_Supplementary-Material [file jiaa046_suppl_supplementary-material.pdf]

BURDEN OF SEVERE RESPIRATORY SYNCYTIAL VIRUS DISEASE IN CHILDREN YOUNGER THAN 2 YEARS OF AGE IN THE SOUTHERN AREA OF BUENOS AIRES, ARGENTINA.

📖 Codebook ▾

📖 Data Dictionary Codebook

08/18/2016 2:57pm

#	Variable / Field Name	Field Label<br>*Field Note*	Field Attributes (Field Type, Validation, Choices, Calculations, etc.)
Instrument: **Epidemiologica2013**			
1	fe_00_hospital_numero	Hospital y Numero	text
2	fe_logo		descriptive
3	fe_00_iniciales	Section Header: *Ficha Epidemiologica*<br>Iniciales	text
4	fe_dni	DNI	text (number, Max: 999)
5	fe_00_tel_fijo	Section Header: *Datos de contacto*<br>Numero de telefono fijo del domicilio	text
6	fe_00_cel_madre	Numero de telefono celular de la madre	text
7	fe_00_cel_padre	Numero de telefono celular del padre	text
8	fe_00_tel_otro	Otro numero de telefono de contacto	text
9	fe_01	Section Header: *Antecedentes personales, familiares y ficha epidemiologica*<br>01 Fecha de consulta<br>*DD-MM-YYYY*	text (date_dmy), Required
10	fe_01_1	01.1 Tipo de cobertura de salud	radio, Required<br>1 N/c<br>2 Obra social/prepaga<br>3 Sin cobertura<br>Custom alignment: RH
11	fe_02	02 Fecha de nacimiento<br>*DD-MM-YYYY*	text (date_dmy), Required
12	fe_03	03 Edad gestacional	text (number), Required
13	fe_04	04 Sexo	radio, Required<br>1 Femenino<br>2 Masculino<br>Custom alignment: RH
14	fe_05	05 Peso al nacer<br>*gramos*	text (number, Min: 600, Max: 6500), Required

15	fe_06_1	06.¿Cuántas veces durante el embarazo la madre consultó al obstreta para seguimiento del mismo?	radio<br>1 Nunca<br>2 Menos de 1 vez por trimestre<br>3 1 vez por trimestre o más y menos de 1 vez por mes<br>4 1 vez por mes o más
16	fe_06_2<br><br>Show the field ONLY if:<br>[fe_06_1] = '4'	Motivo	text
17	fe_06_nc<br><br>Show the field ONLY if:<br>[fe_06_1] = '4'		checkbox<br>1 fe_06_nc___1 N/c
18	fe_07	07 Tipo de parto	radio, Required<br>1 N/c<br>2 Vaginal<br>3 Cesarea<br><br>Custom alignment: RH
19	fe_08	08 Retraso de crecimiento intrauterino	radio, Required<br>1 N/c<br>2 No<br>3 Si<br><br>Custom alignment: RH
20	fe_12	09 Internacion en neonatologia al nacer	radio, Required<br>1 N/c<br>2 No<br>3 Si<br><br>Custom alignment: RH
21	fe_12_1_1<br><br>Show the field ONLY if:<br>[fe_12] = '3'	9.1 Diagnostico de ingreso 1	text
22	fe_12_1_a<br><br>Show the field ONLY if:<br>[fe_12] = '3'	09.1.a N/c	checkbox<br>1 fe_12_1_a___1 N/c
23	fe_12_2<br><br>Show the field ONLY if:<br>[fe_12] = '3'	09.2 Dias de internacion	text (number)
24	fe_12_2_a<br><br>Show the field ONLY if:<br>[fe_12] = '3'	09.2.a N/c	checkbox<br>1 fe_12_2_a___1 N/c

25	fe_12_3<br><br>Show the field ONLY if:<br>[fe_12] = '3'	09.3 Requerimiento de ARM	radio<br>1 N/c<br>2 No<br>3 Si<br><br>Custom alignment: RH
26	fe_12_3_a<br><br>Show the field ONLY if:<br>[fe_12] = '3' and [fe_12_3] = '3'	09.3.a Requerimiento de ARM - Dias	text (number)
27	fe_12_4<br><br>Show the field ONLY if:<br>[fe_12] = '3'	09.4 Requerimiento O2 suplementario al alta de neonatología	radio<br>1 N/c<br>2 No<br>3 Sí<br><br>Custom alignment: RH
28	fe_10	10 Infección perinatal confirmada:	radio, Required<br>1 N/c<br>2 No<br>3 Si,<br><br>Custom alignment: RH
29	fe_10_1<br><br>Show the field ONLY if:<br>[fe_10] = '3'	10.1 Especifique diagnóstico	checkbox<br>1 fe_10_1___1 10.1 HIV<br>2 fe_10_1___2 10.2 Toxoplasmosis<br>3 fe_10_1___3 10.3 Sífilis<br>4 fe_10_1___4 10.4 Chagas<br>5 fe_10_1___5 10.5 CMV<br>6 fe_10_1___6 10.6 Sepsis por estreptococ del grupoB (Streptococcus agalactinae)<br>7 fe_10_1___7 10.7 Otro
30	fe_10_o<br><br>Show the field ONLY if:<br>[fe_10_1(7)] = '1'		text
31	fe_11	11 Lactancia materna exclusiva actual	radio, Required<br>1 N/c<br>2 No<br>3 Si<br><br>Custom alignment: RH

32	fe_12a	12 Alimentación con lactancia materna exclusiva alguna vez	radio<br>1 N/c<br>2 No<br>3 Sí<br><br>Custom alignment: RH
33	fe_12d<br><br>Show the field ONLY if:<br>[fe_12a] = '3'	Duración en meses	text
34	fe_13_a	13 Alimentación con lactancia + complemento:	radio<br>1 N/c<br>2 No<br>3 Sí<br><br>Custom alignment: RH
35	fe_13d<br><br>Show the field ONLY if:<br>[fe_13_a] = '3'	13.1 Duración en meses	text
36	fe_14_a	14 Alimentación sólo con leche de fórmula:	radio<br>1 N/c<br>2 No<br>3 Sí<br><br>Custom alignment: RH
37	fe_14d<br><br>Show the field ONLY if:<br>[fe_14_a] = '3'	14.1 Duración en meses	text
38	fe_15_a	15 Edad de incorporación de leche de vaca y/o derivados:<br>*(en meses)*	text<br>Custom alignment: RH
39	fe_15d		radio<br>1 N/c
40	fe_16_a	16 Edad de incorporación de semisólidos:<br>*(en meses)*	text<br>Custom alignment: RH
41	fe_16d		radio<br>1 N/c
42	fe_15	17 ¿Recibe o recibió suplemento de vitamina D?	radio, Required<br>1 N/c<br>2 No<br>3 Si<br><br>Custom alignment: RH
43	fe_15_d<br><br>Show the field ONLY if:<br>[fe_15] = '3'	17.1 Duración en meses	text (number)

44	fe_16	18¿Recibe o recibió suplemento de vitamina C?	radio, Required<br>1 N/c<br>2 No<br>3 Si<br><br>Custom alignment: RH
45	fe_16_d<br><br>Show the field ONLY if:<br>[fe_16] = '3'	Duración en meses	text (number)
46	fe_19a	19¿Recibe o recibió suplemento de vitamina A?	radio, Required<br>1 N/c<br>2 No<br>3 Si<br><br>Custom alignment: RH
47	fe_19_d<br><br>Show the field ONLY if:<br>[fe_19a] = '3'	Duración en meses	text (number)
48	fe_20a	20¿Recibe o recibió suplemento de sulfato ferroso?	radio, Required<br>1 N/c<br>2 No<br>3 Si<br><br>Custom alignment: RH
49	fe_20_d<br><br>Show the field ONLY if:<br>[fe_20a] = '3'	Duración en meses	text (number)
50	fe_17	Section Header: *Antecedentes inmunológicos*<br>21 Vacunas completas según carnet	radio, Required<br>1 N/c<br>2 No<br>3 Si<br><br>Custom alignment: RH
51	fe_22_bcg	Section Header: *22 Complete según vacuna y número de dosis aplicadas*<br>BCG	radio (Matrix)<br>0 0<br>1 1<br>2 2<br>3 3<br>4 4<br>5 5

52	fe_22_hep_b	Hepatitis B	radio (Matrix)<br>0 0<br>1 1<br>2 2<br>3 3<br>4 4<br>5 5
53	fe_22_sab	Sabín	radio (Matrix)<br>0 0<br>1 1<br>2 2<br>3 3<br>4 4<br>5 5
54	fe_22_pen	Pentavalente (DPT-Hib-HBV)	radio (Matrix)<br>0 0<br>1 1<br>2 2<br>3 3<br>4 4<br>5 5
55	fe_22_trv	Triple viral	radio (Matrix)<br>0 0<br>1 1<br>2 2<br>3 3<br>4 4<br>5 5
56	fe_22_hep_a	Hepatitis A	radio (Matrix)<br>0 0<br>1 1<br>2 2<br>3 3<br>4 4<br>5 5
57	fe_22_13s	Antineumocóccica conjugada 13 serotipos	radio (Matrix)<br>0 0<br>1 1<br>2 2<br>3 3<br>4 4<br>5 5

58	fe_22_11s	Antineumocóccica conjugada 11 serotipos	radio (Matrix)<br>0 0<br>1 1<br>2 2<br>3 3<br>4 4<br>5 5
59	fe_22_gri	Antigripal	radio (Matrix)<br>0 0<br>1 1<br>2 2<br>3 3<br>4 4<br>5 5
60	fe_22_cua	Cuádruple (DPT-Hib)	radio (Matrix)<br>0 0<br>1 1<br>2 2<br>3 3<br>4 4<br>5 5
61	fe_22_sex	Séxtuple acelular (DPaT-Hib-IPV-HB)	radio (Matrix)<br>0 0<br>1 1<br>2 2<br>3 3<br>4 4<br>5 5
62	fe_22_qui	Quintuple acelular (DPaT-Hib-IPV)	radio (Matrix)<br>0 0<br>1 1<br>2 2<br>3 3<br>4 4<br>5 5
63	fe_22_var	Varicela	radio (Matrix)<br>0 0<br>1 1<br>2 2<br>3 3<br>4 4<br>5 5

64	fe_22_roq	Rotavirus Rotateq	radio (Matrix)<br>0 0<br>1 1<br>2 2<br>3 3<br>4 4<br>5 5
65	fe_22_rox	Rotavirus Rotarix	radio (Matrix)<br>0 0<br>1 1<br>2 2<br>3 3<br>4 4<br>5 5
66	fe_22_goc	Antimeningocóccica conjugada C	radio (Matrix)<br>0 0<br>1 1<br>2 2<br>3 3<br>4 4<br>5 5
67	fe_22_135	Antimeningocóccica conjugada tetravalente A,C,Y, W135	radio (Matrix)<br>0 0<br>1 1<br>2 2<br>3 3<br>4 4<br>5 5
68	fe_19	23 Profilaxis con palivizumab	radio, Required<br>1 N/c<br>2 No<br>3 Si<br><br>Custom alignment: RH
69	fe_19_1<br><br>Show the field ONLY if:<br>[fe_19] = '3'	23.1 Número dosis	text (number)
70	fe_19_2<br><br>Show the field ONLY if:<br>[fe_19] = '3'	23.2 Fecha de última dosis<br>*DD-MM-YYYY*	text (date_dmy)
71	fe_24a	24 Vacunas del embarazo:	descriptive

72	fe_24a_1	24.1 Antigripal:	radio<br>1 | Ns/nc<br>2 | No<br>3 | Sí, detalle:<br><br>Custom alignment: RH
73	fe_24_1tr<br><br>Show the field ONLY if:<br>[fe_24a_1] = '3'	24.1.1 Trimestre en el que se aplicó	radio<br>1 | 1°<br>2 | 2°<br>3 | 3°<br><br>Custom alignment: RH
74	fe_24_1fe<br><br>Show the field ONLY if:<br>[fe_24a_1] = '3'	24.1.2 Fecha de aplicación:<br>*(dd/mm/aa)*	text (date_dmy)
75	fe_24a_2	24.2 Triple bacteriana acelular:	radio<br>1 | Ns/nc<br>2 | No<br>3 | Sí, detalle:<br><br>Custom alignment: RH
76	fe_24_2tr<br><br>Show the field ONLY if:<br>[fe_24a_2] = '3'	24.2.1 Trimestre en el que se aplicó	radio<br>1 | 1°<br>2 | 2°<br>3 | 3°<br><br>Custom alignment: RH
77	fe_24_2fe<br><br>Show the field ONLY if:<br>[fe_24a_2] = '3'	24.2.2 Fecha de aplicación:<br>*(dd/mm/aa)*	text (date_dmy)
78	fe_24a_3	24.3 Doble adultos:	radio<br>1 | Ns/nc<br>2 | No<br>3 | Sí, detalle:<br><br>Custom alignment: RH
79	fe_24_3tr<br><br>Show the field ONLY if:<br>[fe_24a_3] = '3'	24.3.1 Trimestre en el que se aplicó	radio<br>1 | 1°<br>2 | 2°<br>3 | 3°<br><br>Custom alignment: RH
80	fe_24_3fe<br><br>Show the field ONLY if:<br>[fe_24a_3] = '3'	24.3.2 Fecha de aplicación:<br>*(dd/mm/aa)*	text (date_dmy)

<table>
<tr><td>81</td><td>fe_24a_4</td><td>24.4 Antitetánica</td><td>radio<table><tr><td>1</td><td>Ns/nc</td></tr><tr><td>2</td><td>No</td></tr><tr><td>3</td><td>Sí, detalle:</td></tr></table>Custom alignment: RH</td></tr>
<tr><td>82</td><td>fe_24_4tr
Show the field ONLY if: [fe_24a_4] = '3'</td><td>24.4.1 Trimestre en el que se aplicó</td><td>radio<table><tr><td>1</td><td>1°</td></tr><tr><td>2</td><td>2°</td></tr><tr><td>3</td><td>3°</td></tr></table>Custom alignment: RH</td></tr>
<tr><td>83</td><td>fe_24_4fe
Show the field ONLY if: [fe_24a_4] = '3'</td><td>24.4.2 Fecha de aplicación:
(dd/mm/aa)</td><td>text (date_dmy)</td></tr>
<tr><td>84</td><td>fe_20</td><td>Section Header: Antecedentes patologicos
25 Peso actual
gramos</td><td>text (number, Min: 1000, Max: 25000), Required</td></tr>
<tr><td>85</td><td>fe_21</td><td>26 Peso a los 3 meses de vida (si corresponde)
gramos</td><td>text (number, Min: 1500, Max: 10000)</td></tr>
<tr><td>86</td><td>fe_22</td><td>27 Peso a los 6 meses (si corresponde)
gramos</td><td>text (number, Min: 3000, Max: 15000)</td></tr>
<tr><td>87</td><td>fe_28a</td><td>28 Broncoespasmos a repetición (más de 3 episodios en el último año en los que requirió uso de salbutamol):</td><td>radio<table><tr><td>1</td><td>N/c</td></tr><tr><td>2</td><td>No</td></tr><tr><td>3</td><td>Sí</td></tr></table>Custom alignment: RH</td></tr>
<tr><td>88</td><td>fe_29a</td><td>29 ¿Recibe en forma continua corticoides inhalatorios desde hace más de 2 meses (budesonide/fluticasona)?</td><td>radio<table><tr><td>1</td><td>N/c</td></tr><tr><td>2</td><td>No</td></tr><tr><td>3</td><td>Sí</td></tr></table>Custom alignment: RH</td></tr>
<tr><td>89</td><td>fe_tit</td><td>Marque si presenta alguna de las siguientes patologías:</td><td>descriptive</td></tr>
<tr><td>90</td><td>fe_25</td><td>30 Atopia (rinitis alérgica, dermatitis atópica, eccema)</td><td>radio, Required<table><tr><td>1</td><td>N/c</td></tr><tr><td>2</td><td>No</td></tr><tr><td>3</td><td>Si</td></tr></table>Custom alignment: RH</td></tr>
<tr><td>91</td><td>fe_26</td><td>31 Fibrosis quistica</td><td>radio, Required<table><tr><td>1</td><td>N/c</td></tr><tr><td>2</td><td>No</td></tr><tr><td>3</td><td>Si</td></tr></table>Custom alignment: RH</td></tr>
</table>

92	fe_27	32 Cardiopatia congenita	radio, Required<br>1 N/c<br>2 No<br>3 Si<br><br>Custom alignment: RH
93	fe_27_a<br><br>Show the field ONLY if:<br>[fe_27] = '3'	¿Cuál?	text
94	fe_27_1<br><br>Show the field ONLY if:<br>[fe_27] = '3'	32.1 Cardiopatia congenita - Correccion quirurgica	radio<br>1 N/c<br>2 No<br>3 Si<br><br>Custom alignment: RH
95	fe_28	33 Sindrome de Down	radio, Required<br>1 N/c<br>2 No<br>3 Si<br><br>Custom alignment: RH
96	fe_29	34 Inmunodeficiencia	radio, Required<br>1 N/c<br>2 No<br>3 Si<br><br>Custom alignment: RH
97	fe_29e<br><br>Show the field ONLY if:<br>[fe_29] = '3'	Especifique	text
98	fe_30	35 Patologia neorologica	radio, Required<br>1 N/c<br>2 No<br>3 Si<br><br>Custom alignment: RH
99	fe_30_a<br><br>Show the field ONLY if:<br>[fe_30] = '3'	Diagnostico	text
100	fe_31	36 Patologia Hematologica	radio, Required<br>1 N/c<br>2 No<br>3 Si<br><br>Custom alignment: RH
101	fe_31_a<br><br>Show the field ONLY if:<br>[fe_31] = '3'	Diagnostico	text

102	fe_32	37 ¿Recibio tratamiento antibiotico en los últimos 3 meses?	radio, Required<br>1 N/c<br>2 No<br>3 Si<br><br>Custom alignment: RH
103	fe_32_1<br><br>Show the field ONLY if:<br>[fe_32] = '3'	37. 1 Causa	text
104	fe_32_1_a<br><br>Show the field ONLY if:<br>[fe_32] = '3'	37.1.a N/c	checkbox<br>1 fe_32_1_a___1 N/c
105	fe_32_2<br><br>Show the field ONLY if:<br>[fe_32] = '3'	37.2 Antibiótico recibido	text
106	fe_32_2_a<br><br>Show the field ONLY if:<br>[fe_32] = '3'	37.2.a N/c	checkbox<br>1 fe_32_2_a___1 N/c
107	fe_32_3<br><br>Show the field ONLY if:<br>[fe_32] = '3'	37.3 Dias totales de tratamiento	text (number)
108	fe_32_3_a<br><br>Show the field ONLY if:<br>[fe_32] = '3'	37.3.a N/c	checkbox<br>1 fe_32_3_a___1 N/c
109	fe_33	38 Otra condicion patologica de base:	text
110	fe_39	39 Internaciones previas	radio, Required<br>1 N/c<br>2 No<br>3 Si<br><br>Custom alignment: RH
111	fe_tit39<br><br>Show the field ONLY if:<br>[fe_39] = '3'	Detallar comenzando por la última internación:	descriptive
112	fe_39_1<br><br>Show the field ONLY if:<br>[fe_39] = '3'	39.1 Internacion 1	descriptive
113	fe_39_1_a<br><br>Show the field ONLY if:<br>[fe_39] = '3'	39.1.a Internacion 1 - Edad	text (number)
114	fe_39_1_b<br><br>Show the field ONLY if:<br>[fe_39] = '3'	39.1.b Internacion 1 - Diagnostico	text

115	fe_39_1_c<br><br>Show the field ONLY if:<br>[fe_39] = '3'	39.1.c Internacion 1 - Tipo	radio<br>1 Sala de pediatria<br>2 UCIN<br>3 UCIP<br><br>Custom alignment: RH
116	fe_39_1_d_sala<br><br>Show the field ONLY if:<br>[fe_39_1_c] = '1'	39.1.d_sala Internacion 1 - Duracion Sala	text (number)
117	fe_39_1_d_ucin<br><br>Show the field ONLY if:<br>[fe_39_1_c] = '2'	39.1.d_UCIN Internacion 1 - Duracion UCIN	text (number)
118	fe_39_1_d_ucip<br><br>Show the field ONLY if:<br>[fe_39_1_c] = '3'	39.1.d_UCIP Internacion 1 - Duracion UCIP	text (number)
119	fe_39_1_e<br><br>Show the field ONLY if:<br>[fe_39] = '3'	39.1.e Internacion 1 - Oxigeno	radio<br>1 No<br>2 Si<br><br>Custom alignment: RH
120	fe_39_1_f<br><br>Show the field ONLY if:<br>[fe_39_1_e] = '2'	39.1.f Internacion 1 - Oxigeno - Dias	text (number)
121	fe_39_2<br><br>Show the field ONLY if:<br>[fe_39_1_a] <>" or [fe_39_1_b] =<>"	39.2 Internacion 2	descriptive
122	fe_39_2_a<br><br>Show the field ONLY if:<br>[fe_39_1_a]<>" or [fe_39_1_b]<>"	39.2.a Internacion 2 - Edad	text (number)
123	fe_39_2_b<br><br>Show the field ONLY if:<br>[fe_39_1_a]<>" or [fe_39_1_b]<>"	39.2.b Internacion 2 - Diagnostico	text
124	fe_39_2_c<br><br>Show the field ONLY if:<br>[fe_39_1_a]<>" or [fe_39_1_b]<>"	39.2.c Internacion 2 - Tipo	radio<br>1 Sala de pediatria<br>2 UCIN<br>3 UCIP<br><br>Custom alignment: RH
125	fe_39_2_d_sala<br><br>Show the field ONLY if:<br>[fe_39_2_c] = '1'	39.2.d_sala Internacion 2 - Duracion Sala	text (number)
126	fe_39_2_d_ucin<br><br>Show the field ONLY if:<br>[fe_39_2_c] = '2'	39.2.d_UCIN Internacion 2 - Duracion UCIN	text (number)

127	fe_39_2_d_ucip<br><br>Show the field ONLY if:<br>[fe_39_2_c] = '3'	39.2.d_UCIP Internacion 2 - Duracion UCIP	text (number)
128	fe_39_2_e<br><br>Show the field ONLY if:<br>[fe_39_1_a]<>" or [fe_39_1_b]<>"	39.2.e Internacion 2 - Oxigeno	radio<br>1 No<br>2 Si<br><br>Custom alignment: RH
129	fe_39_2_f<br><br>Show the field ONLY if:<br>[fe_39_2_e] = '2'	39.2.f Internacion 2 - Oxigeno - Dias	text (number)
130	fe_39_3<br><br>Show the field ONLY if:<br>[fe_39_2_a]<>" or [fe_39_2_b]<>"	39.3 Internacion 3	descriptive
131	fe_39_3_a<br><br>Show the field ONLY if:<br>[fe_39_2_a]<>" or [fe_39_2_b]<>"	39.3.a Internacion 3 - Edad	text (number)
132	fe_39_3_b<br><br>Show the field ONLY if:<br>[fe_39_2_a]<>" or [fe_39_2_b]<>"	39.3.b Internacion 3 - Diagnostico	text
133	fe_39_3_c<br><br>Show the field ONLY if:<br>[fe_39_2_a]<>" or [fe_39_2_b]<>"	39.3.c Internacion 3 - Tipo	radio<br>1 Sala de pediatria<br>2 UCIN<br>3 UCIP<br><br>Custom alignment: RH
134	fe_39_3_d_sala<br><br>Show the field ONLY if:<br>[fe_39_3_c] = '1'	39.3.d_sala Internacion 3 - Duracion Sala	text (number)
135	fe_39_3_d_ucin<br><br>Show the field ONLY if:<br>[fe_39_3_c] = '2'	39.3.d_UCIN Internacion 3 - Duracion UCIN	text (number)
136	fe_39_3_d_ucip<br><br>Show the field ONLY if:<br>[fe_39_3_c] = '3'	39.3.d_UCIP Internacion 3 - Duracion UCIP	text (number)
137	fe_39_3_e<br><br>Show the field ONLY if:<br>[fe_39_2_a]<>" or [fe_39_2_b]<>"	39.3.e Internacion 3 - Oxigeno	radio<br>1 No<br>2 Si<br><br>Custom alignment: RH
138	fe_39_3_f<br><br>Show the field ONLY if:<br>[fe_39_3_e] = '2'	39.3.f Internacion 3 - Oxigeno - Dias	text (number)

139	fe_39_4<br><br>Show the field ONLY if:<br>[fe_39_3_a]<>" or [fe_39_3_b]<>"	39.4 Internacion 4	descriptive
140	fe_39_4_a<br><br>Show the field ONLY if:<br>[fe_39_3_a]<>" or [fe_39_3_b]<>"	39.4.a Internacion 4 - Edad	text (number)
141	fe_39_4_b<br><br>Show the field ONLY if:<br>[fe_39_3_a]<>" or [fe_39_3_b]<>"	39.4.b Internacion 4 - Diagnostico	text
142	fe_39_4_c<br><br>Show the field ONLY if:<br>[fe_39_3_a]<>" or [fe_39_3_b]<>"	39.4.c Internacion 4 - Tipo	radio<br>1 Sala de pediatria<br>2 UCIN<br>3 UCIP<br><br>Custom alignment: RH
143	fe_39_4_d_sala<br><br>Show the field ONLY if:<br>[fe_39_4_c] = '1'	39.4.d_sala Internacion 4 - Duracion Sala	text (number)
144	fe_39_4_d_ucin<br><br>Show the field ONLY if:<br>[fe_39_4_c] = '2'	39.4.d_UCIN Internacion 4 - Duracion UCIN	text (number)
145	fe_39_4_d_ucip<br><br>Show the field ONLY if:<br>[fe_39_4_c] = '3'	39.4.d_UCIP Internacion 4 - Duracion UCIP	text (number)
146	fe_39_4_e<br><br>Show the field ONLY if:<br>[fe_39_3_a]<>" or [fe_39_3_b]<>"	39.4.e Internacion 4 - Oxigeno	radio<br>1 No<br>2 Si<br><br>Custom alignment: RH
147	fe_39_4_f<br><br>Show the field ONLY if:<br>[fe_39_4_e] = '2'	39.4.f Internacion 4 - Oxigeno - Dias	text (number)
148	fe_39_5<br><br>Show the field ONLY if:<br>[fe_39_4_a]<>" or [fe_39_4_b]<>"	39.5. Internacion 5	descriptive
149	fe_39_5_a<br><br>Show the field ONLY if:<br>[fe_39_4_a]<>" or [fe_39_4_b]<>"	39.5.a Internacion 5 - Edad	text (number)
150	fe_39_5_b<br><br>Show the field ONLY if:<br>[fe_39_4_a]<>" or [fe_39_4_b]<>"	39.5.b Internacion 5 - Diagnostico	text

151	fe_39_5_c<br><br>Show the field ONLY if:<br>[fe_39_4_a]<>" or [fe_39_4_b]<>"	39.5.c Internacion 5 - Tipo	radio<br>1 Sala de pediatria<br>2 UCIN<br>3 UCIP<br><br>Custom alignment: RH
152	fe_39_5_d_sala<br><br>Show the field ONLY if:<br>[fe_39_5_c] = '1'	39.5.d_sala Internacion 5 - Duracion Sala	text (number)
153	fe_39_5_d_ucin<br><br>Show the field ONLY if:<br>[fe_39_5_c] = '2'	39.5.d_UCIN Internacion 5 - Duracion UCIN	text (number)
154	fe_39_5_d_ucip<br><br>Show the field ONLY if:<br>[fe_39_5_c] = '3'	39.5.d_UCIP Internacion 5 - Duracion UCIP	text (number)
155	fe_39_5_e<br><br>Show the field ONLY if:<br>[fe_39_4_a]<>" or [fe_39_4_b]<>"	39.5.e Internacion 5 - Oxigeno	radio<br>1 No<br>2 Si<br><br>Custom alignment: RH
156	fe_39_5_f<br><br>Show the field ONLY if:<br>[fe_39_5_e] = '2'	39.5.f Internacion 5 - Oxigeno - Dias	text (number)
157	fe_40_0	Section Header: *Datos epidemiológicos:*<br>Domicilio:	descriptive
158	fe_35_a	40.a Localidad	text, Required
159	fe_35_b	40.b Partido	text, Required
160	fe_36	41 Ubicacion	radio, Required<br>1 Urbana<br>2 Rural<br>3 Villa de emergencia<br><br>Custom alignment: RH
161	fe_37	42 Condicion mayoritaria en las calles del barrio	radio, Required<br>1 Tierra<br>2 Asfalto - Empedrado<br><br>Custom alignment: RH
162	fe_38	43 Tiempo de viaje estimado desde su casa hasta el hospital	radio<br>1 menos media hora<br>2 entre 30 minutos y 1 hora<br>3 entre 1 y 2 horas<br>4 mas de 2 horas
163	fe_44_0	Vivienda	descriptive

164	fe_39_v	44 Vivienda - Material	checkbox, Required<br>1 fe_39_v___1 chapa<br>2 fe_39_v___2 madera<br>3 fe_39_v___3 adobe<br>4 fe_39_v___4 cemento<br>5 fe_39_v___5 otro<br><br>Custom alignment: RH
165	fe_39_a<br><br>Show the field ONLY if:<br>[fe_39_v(5)] = '1'	44.a Vivienda - Material - Otro	text
166	fe_40	45 Vivienda - Piso	radio, Required<br>1 tierra<br>2 cemento<br>3 madera<br>4 otro<br><br>Custom alignment: RH
167	fe_40_a<br><br>Show the field ONLY if:<br>[fe_40] = '4'	45.a Vivienda - Piso - Otro	text
168	fe_41	46 Vivienda - Agua	radio, Required<br>1 de pozo<br>2 corriente de red<br>3 otro<br><br>Custom alignment: RH
169	fe_41_a<br><br>Show the field ONLY if:<br>[fe_41] = '3'	46.a Vivienda - Agua - Otro	text
170	fe_42	47 Vivienda - Excretas	radio, Required<br>1 pozo ciego<br>2 cloacas<br>3 otro<br><br>Custom alignment: RH
171	fe_42_a<br><br>Show the field ONLY if:<br>[fe_42] = '3'	47.a Vivienda - Excretas - Otro	text
172	fe_43	48 Vivienda - Calefaccion	radio, Required<br>1 gas<br>2 kerosene<br>3 madera/leña/carbon<br>4 otro<br><br>Custom alignment: RH

173	fe_43_a<br><br>Show the field ONLY if:<br>[fe_43] = '4'	48.a Vivienda - Calefaccion - Otro	text
174	fe_44	49 Vivienda - Número de habitaciones (excluyendo baño y cocina):	text (number), Required
175	fe_45	50 Número de convivientes:	text (number)
176	fe_46	51 ¿Existen convivientes que habitualmente fuman dentro de la casa?	radio, Required<br>1 N/c<br>2 No<br>3 Si
177	fe_46a<br><br>Show the field ONLY if:<br>[fe_46] = '3'	51.a ¿Cuántos?	text (number)
178	fe_52_1	52 Convivientes que habitualmente fuman, pero fuera de la casa:	radio, Required<br>1 N/c<br>2 No<br>3 Si
179	fe_52a<br><br>Show the field ONLY if:<br>[fe_52_1] = '3'	52.a ¿Cuántos?	text (number)
180	fe_53_1	53 Vivienda - Número de niños menores de 10 años convivientes:	text (number), Required
181	fe_con	Contactos	descriptive
182	fe_54_1	54 Concurre a guarderia/jardín maternal duranate más de 4 horas diarias:	radio, Required<br>1 N/c<br>2 No<br>3 Si<br><br>Custom alignment: RH
183	fe_50	55 Animales domésticos	radio, Required<br>1 N/c<br>2 No<br>3 Si<br><br>Custom alignment: RH
184	fe_50_a<br><br>Show the field ONLY if:<br>[fe_50] = '3'	55.a Animales - Detalle	checkbox<br>1 fe_50_a___1 Perro<br>3 fe_50_a___3 Gato<br>4 fe_50_a___4 Otros

185	fe_51	Section Header: *Antecedentes familiares*<br>56. Cantidad de hermanos del niño:	dropdown, Required<br>13 N/c<br>0 0<br>1 1<br>2 2<br>3 3<br>4 4<br>5 5<br>6 6<br>7 7<br>8 8<br>9 9<br>10 10<br>11 11<br>12 12
186	fe_56_0<br>Show the field ONLY if: [fe_51] = '1' or [fe_51] = '2' or [fe_51] = '3' or [fe_51] = '4' or [fe_51] = '5' or [fe_51] = '6' or [fe_51] = '7' or [fe_51] = '8' or [fe_51] = '9' or [fe_51] = '10' or [fe_51] = '11' or [fe_51] = '12'	Si tiene hermanos, ¿padecen alguna de las siguientes patologías diagnosticadas por un médico?	descriptive
187	fe_51_1<br>Show the field ONLY if: [fe_51] = '1' or [fe_51] = '2' or [fe_51] = '3' or [fe_51] = '4' or [fe_51] = '5' or [fe_51] = '6' or [fe_51] = '7' or [fe_51] = '8' or [fe_51] = '9' or [fe_51] = '10' or [fe_51] = '11' or [fe_51] = '12'	56.1 Asma	radio<br>1 N/c<br>2 No<br>3 Si<br>Custom alignment: RH
188	fe_51_1_a<br>Show the field ONLY if: [fe_51_1] = '3'	56.1.a Asma - ¿Cuántos?	text (number)
189	fe_51_2<br>Show the field ONLY if: [fe_51] = '1' or [fe_51] = '2' or [fe_51] = '3' or [fe_51] = '4' or [fe_51] = '5' or [fe_51] = '6' or [fe_51] = '7' or [fe_51] = '8' or [fe_51] = '9' or [fe_51] = '10' or [fe_51] = '11' or [fe_51] = '12'	56.2 Atopia	radio<br>1 N/c<br>2 No<br>3 Si<br>Custom alignment: RH
190	fe_51_2_a<br>Show the field ONLY if: [fe_51_2] = '3'	56.2.a Atopia - ¿Cuántos?	text (number)

191	fe_52	Section Header: *Datos de la madre*<br>57 Madre - Fecha de nacimiento<br>*DD-MM-YYYY*	text (date_dmy)
192	fe_52_a	57.a N/c	checkbox<br>1 fe_52_a___1 N/c
193	fe_53	58 Peso manterno antes del embarazo<br>*Kilogramos*	text (number, Min: 35, Max: 150)
194	fe_53_a	58.a N/c	checkbox<br>1 fe_53_a___1 N/c
195	fe_54	59 Talla materna<br>*centímetros*	text (number, Min: 110, Max: 200)
196	fe_54_a	59.a N/c	checkbox<br>1 fe_54_a___1 N/c
197	fe_55	60 Cuantos kilos aumento en el embarazo	text (number, Min: 0, Max: 30)
198	fe_55_a	60.a N/c	checkbox<br>1 fe_55_a___1 N/c
199	fe_56	61 Peso manterno actual<br>*Kilogramos*	text (number, Min: 35, Max: 150)
200	fe_56_a	61.a N/c	checkbox<br>1 fe_56_a___1 N/c
201	fe_57	62 ¿Cuál es el nivel de educación más alto que ha alcanzado?	radio<br>1 Analfabeta<br>2 Alfabeta sin educacion formal<br>3 Primaria completa<br>4 Primaria incompleta<br>5 Secundaria completa<br>6 Secundaria incompleta<br>7 Terciario/Universitario completo<br>8 Terciario/Universitario incompleto<br>9 n/c
202	fe_58	63 ¿Cuál es el ingreso promedio familiar mensual en pesos?	radio, Required<br>1 N/c<br>2 < 500<br>3 500-1000<br>4 1000-2000<br>5 2000-3000<br>6 > 3000
203	fe_59	64 ¿Fumó usted durante embarazo?	radio, Required<br>1 N/c<br>2 No<br>3 Si<br>Custom alignment: RH

204	fe_59_1<br><br>Show the field ONLY if: [fe_59] = '3'	64.1 ¿Durante cuánto tiempo fumó durante su embarazo?<br>*meses*	dropdown<br>1 | 1<br>2 | 2<br>3 | 3<br>4 | 4<br>5 | 5<br>6 | 6<br>7 | 7<br>8 | 8<br>9 | 9<br>10 | n/c
205	fe_59_2<br><br>Show the field ONLY if: [fe_59] = '3'	64.2 ¿Cuántos cigarrillos por dia?<br>*cigarrillos*	text
206	fe_60	65 ¿Fuma actualmente?	radio, Required<br>1 | N/c<br>2 | No<br>3 | Si<br><br>Custom alignment: RH
207	fe_60_1<br><br>Show the field ONLY if: [fe_60] = '3'	65.1 ¿Cuántos cigarrillos por dia?<br>*cigarrillos*	text
208	fe_61	66 ¿Alguna vez su médico le diagnosticó asma?	radio, Required<br>1 | N/c<br>2 | No<br>3 | Si<br><br>Custom alignment: RH
209	fe_62	67 ¿Usó en el último año Salbutamol/Ventolín en aerosol?	radio, Required<br>1 | N/c<br>2 | No<br>3 | Si<br><br>Custom alignment: RH
210	fe_63	68 ¿Utiliza corticoides en aerosol (budesonide, fluticasona, seretide)?	radio, Required<br>1 | N/c<br>2 | No<br>3 | Si<br><br>Custom alignment: RH
211	fe_69_tit	69 Las siguientes preguntas se refieren a su alimentación durante el último trimestre del embarazo:	descriptive

212	fe_69_ver	Section Header: *¿Con qué frecuencia consumió las siguientes comidas?*<br>Verduras	radio (Matrix)<br>1 Nunca, o menos de una vez al mes<br>2 1 a 3 días al mes<br>3 1 a 3 días a la semana<br>4 4 a 7 días a la semana
213	fe_69_fru	Frutas	radio (Matrix)<br>1 Nunca, o menos de una vez al mes<br>2 1 a 3 días al mes<br>3 1 a 3 días a la semana<br>4 4 a 7 días a la semana
214	fe_69_pap	Papa, Batata	radio (Matrix)<br>1 Nunca, o menos de una vez al mes<br>2 1 a 3 días al mes<br>3 1 a 3 días a la semana<br>4 4 a 7 días a la semana
215	fe_69_arr	Arroz, Pasta	radio (Matrix)<br>1 Nunca, o menos de una vez al mes<br>2 1 a 3 días al mes<br>3 1 a 3 días a la semana<br>4 4 a 7 días a la semana
216	fe_69_pan	Pan	radio (Matrix)<br>1 Nunca, o menos de una vez al mes<br>2 1 a 3 días al mes<br>3 1 a 3 días a la semana<br>4 4 a 7 días a la semana
217	fe_69_fac	Facturas, Tortas	radio (Matrix)<br>1 Nunca, o menos de una vez al mes<br>2 1 a 3 días al mes<br>3 1 a 3 días a la semana<br>4 4 a 7 días a la semana
218	fe_69_gal	Galletitas dulces, masas, golosinas	radio (Matrix)<br>1 Nunca, o menos de una vez al mes<br>2 1 a 3 días al mes<br>3 1 a 3 días a la semana<br>4 4 a 7 días a la semana
219	fe_69_sna	Snacks (papas fritas, palitos, chizitos, 3D,etc.)	radio (Matrix)<br>1 Nunca, o menos de una vez al mes<br>2 1 a 3 días al mes<br>3 1 a 3 días a la semana<br>4 4 a 7 días a la semana

220	fe_69_fri	Fritos	radio (Matrix)<br>1 Nunca, o menos de una vez al mes<br>2 1 a 3 días al mes<br>3 1 a 3 días a la semana<br>4 4 a 7 días a la semana
221	fe_69_roj	Carnes rojas	radio (Matrix)<br>1 Nunca, o menos de una vez al mes<br>2 1 a 3 días al mes<br>3 1 a 3 días a la semana<br>4 4 a 7 días a la semana
222	fe_69_pez	Pescados	radio (Matrix)<br>1 Nunca, o menos de una vez al mes<br>2 1 a 3 días al mes<br>3 1 a 3 días a la semana<br>4 4 a 7 días a la semana
223	fe_69_pol	Pollo	radio (Matrix)<br>1 Nunca, o menos de una vez al mes<br>2 1 a 3 días al mes<br>3 1 a 3 días a la semana<br>4 4 a 7 días a la semana
224	fe_69_msa	Section Header: *¿Con qué frecuencia consumió las siguientes bebidas?*<br>Mate (sin azúcar o con edulcorante)	radio (Matrix)<br>1 Nunca o menos de 1 vez al mes<br>2 1 a 3 días al mes<br>3 1 a 3 días a la semana<br>4 4 a 7 días a la semana
225	fe_69_mca	Mate (con azúcar)	radio (Matrix)<br>1 Nunca o menos de 1 vez al mes<br>2 1 a 3 días al mes<br>3 1 a 3 días a la semana<br>4 4 a 7 días a la semana
226	fe_69_tsa	Té/café (sin azúcar o con edulcorante)	radio (Matrix)<br>1 Nunca o menos de 1 vez al mes<br>2 1 a 3 días al mes<br>3 1 a 3 días a la semana<br>4 4 a 7 días a la semana
227	fe_69_tca	Té/ café (con azúcar)	radio (Matrix)<br>1 Nunca o menos de 1 vez al mes<br>2 1 a 3 días al mes<br>3 1 a 3 días a la semana<br>4 4 a 7 días a la semana

228	fe_69_cer	Cerveza	radio (Matrix)<br>1 Nunca o menos de 1 vez al mes<br>2 1 a 3 días al mes<br>3 1 a 3 días a la semana<br>4 4 a 7 días a la semana
229	fe_69_vin	Vino	radio (Matrix)<br>1 Nunca o menos de 1 vez al mes<br>2 1 a 3 días al mes<br>3 1 a 3 días a la semana<br>4 4 a 7 días a la semana
230	fe_69_ape	Aperitivo (Gancia, Fernet)	radio (Matrix)<br>1 Nunca o menos de 1 vez al mes<br>2 1 a 3 días al mes<br>3 1 a 3 días a la semana<br>4 4 a 7 días a la semana
231	fe_69_bbl	Bebidas blancas (vodka, ron, whisky)	radio (Matrix)<br>1 Nunca o menos de 1 vez al mes<br>2 1 a 3 días al mes<br>3 1 a 3 días a la semana<br>4 4 a 7 días a la semana
232	fe_69_jnd	Gaseosas o jugos no dietéticos	radio (Matrix)<br>1 Nunca o menos de 1 vez al mes<br>2 1 a 3 días al mes<br>3 1 a 3 días a la semana<br>4 4 a 7 días a la semana
233	fe_69_gjd	Gaseosas o jugos dietéticos	radio (Matrix)<br>1 Nunca o menos de 1 vez al mes<br>2 1 a 3 días al mes<br>3 1 a 3 días a la semana<br>4 4 a 7 días a la semana
234	fe_65	70 ¿Padece alguna enfermedad cronica?	radio, Required<br>1 N/c<br>2 No<br>3 Si<br><br>Custom alignment: RH
235	fe_65_1<br><br>Show the field ONLY if:<br>[fe_65] = '3'	70.1 Enfermedad cronica - Detalle	text

<table>
<tr><td>236</td><td>fe_66</td><td>71 ¿Tuvo hipertension en el embarazo?</td><td>radio, Required<table><tr><td>1</td><td>N/c</td></tr><tr><td>2</td><td>No</td></tr><tr><td>3</td><td>Si</td></tr></table>Custom alignment: RH</td></tr>
<tr><td>237</td><td>fe_72</td><td>72 ¿Tuvo diabetes o intolerancia a la glucosa en el embarazo?</td><td>radio, Required<table><tr><td>1</td><td>N/c</td></tr><tr><td>2</td><td>No</td></tr><tr><td>3</td><td>Si</td></tr></table>Custom alignment: RH</td></tr>
<tr><td>238</td><td>fe_73</td><td>73 ¿Tuvo trigliceridos aumentados en el embarazo?</td><td>radio, Required<table><tr><td>1</td><td>N/c</td></tr><tr><td>2</td><td>No</td></tr><tr><td>3</td><td>Si</td></tr></table>Custom alignment: RH</td></tr>
<tr><td>239</td><td>fe_74_h</td><td>74 ¿Tiene HIV?</td><td>radio, Required<table><tr><td>1</td><td>N/c</td></tr><tr><td>2</td><td>No</td></tr><tr><td>3</td><td>Si</td></tr></table>Custom alignment: RH</td></tr>
<tr><td>240</td><td>fe_75_i</td><td>75 ¿Durante el último trimestre de embarazo, tuvo alguna infección?</td><td>radio<table><tr><td>1</td><td>N/c</td></tr><tr><td>2</td><td>No</td></tr><tr><td>3</td><td>Sí</td></tr></table>Custom alignment: RH</td></tr>
<tr><td>241</td><td>fe_75_1

Show the field ONLY if:
[fe_75_i] = '3'</td><td>75.1 Detalle cuál:</td><td>checkbox<table><tr><td>1</td><td>fe_75_1___1</td><td>Infección urinaria</td></tr><tr><td>2</td><td>fe_75_1___2</td><td>Hisopado/ Cultivo Streptococcus agalactiae (+) semana 30-35</td></tr><tr><td>3</td><td>fe_75_1___3</td><td>Rotura prematura de membranas (bolsa)</td></tr><tr><td>4</td><td>fe_75_1___4</td><td>Gripe</td></tr><tr><td>5</td><td>fe_75_1___5</td><td>Infección respiratoria alta (resfrío, catarro, sinusitis)</td></tr><tr><td>6</td><td>fe_75_1___6</td><td>Neumonía</td></tr><tr><td>7</td><td>fe_75_1___7</td><td>Neumonitis</td></tr><tr><td>8</td><td>fe_75_1___8</td><td>Bronquitis</td></tr><tr><td>9</td><td>fe_75_1___9</td><td>Otras</td></tr></table></td></tr>
</table>

242	fe_75_o<br><br>Show the field ONLY if:<br>[fe_75_1(9)] = '1'	Especifique	text
243	fe_69_1<br><br>Show the field ONLY if:<br>[fe_75_i] = '3'	75.2 En caso afirmativo, especifique qué antibiotico/ antiviral recibió	text
244	fe_75_2nc<br><br>Show the field ONLY if:<br>[fe_75_i] = '3'	75.2 Contestó	radio<br>1 Sí<br>2 No<br><br>Custom alignment: RH
245	fe_69_3<br><br>Show the field ONLY if:<br>[fe_75_i] = '3'	75.3 ¿En qué forma lo recibió?	radio<br>1 Oral<br>2 Intravenoso<br>3 N/c
246	fe_69_4<br><br>Show the field ONLY if:<br>[fe_75_i] = '3'	75.4 ¿Requirió internación?	checkbox<br>1 fe_69_4___1 Internacion durante el embarazo<br>2 fe_69_4___2 Internacion durante el parto<br>3 fe_69_4___3 Ambulatorio
247	fe_76	76 ¿Consumió analgésicos/ antitérmicos durante el embarazo?	radio<br>1 N/c<br>2 No<br>3 Si<br><br>Custom alignment: RH
248	fe_76_para<br><br>Show the field ONLY if:<br>[fe_76] = '3'	Section Header: *76.1 Medicamentos*<br>Paracetamol	radio (Matrix)<br>1 Sí<br>2 No<br>3 N/c
249	fe_76_ibup<br><br>Show the field ONLY if:<br>[fe_76] = '3'	Ibuprofeno	radio (Matrix)<br>1 Sí<br>2 No<br>3 N/c
250	fe_76_dipi<br><br>Show the field ONLY if:<br>[fe_76] = '3'	Dipirona	radio (Matrix)<br>1 Sí<br>2 No<br>3 N/c
251	fe_76_aspi<br><br>Show the field ONLY if:<br>[fe_76] = '3'	Aspirina	radio (Matrix)<br>1 Sí<br>2 No<br>3 N/c

252	fe_76_dicl<br><br>Show the field ONLY if:<br>[fe_76] = '3'	Diclofenac	radio (Matrix)<br>1 Sí<br>2 No<br>3 N/c
253	fe_76_par_tri<br><br>Show the field ONLY if:<br>[fe_76_para] = '1'	Paracetamol	checkbox<br>1 fe_76_par_tri___1 Primer trimestre<br>2 fe_76_par_tri___2 Segundo trimestre<br>3 fe_76_par_tri___3 Tercer trimestre
254	fe_76_par1_vez<br><br>Show the field ONLY if:<br>[fe_76_par_tri(1)] = '1'	Paracetamol: Primer trimestre<br>*vez/veces*	dropdown<br>1 1<br>2 2<br>3 3<br>4 4<br>5 5<br>6 6<br>7 7<br>8 8<br>9 9<br>10 10<br>11 >10
255	fe_76_par2_vez<br><br>Show the field ONLY if:<br>[fe_76_par_tri(2)] = '1'	Paracetamol: Segundo trimestre<br>*vez/veces*	dropdown<br>1 1<br>2 2<br>3 3<br>4 4<br>5 5<br>6 6<br>7 7<br>8 8<br>9 9<br>10 10<br>11 >10

256	fe_76_par3_vez<br><br>Show the field ONLY if:<br>[fe_76_par_tri(3)] = '1'	Paracetamol: Tercer trimestre<br>*vez/veces*	dropdown<br>1 — 1<br>2 — 2<br>3 — 3<br>4 — 4<br>5 — 5<br>6 — 6<br>7 — 7<br>8 — 8<br>9 — 9<br>10 — 10<br>11 — >10
257	fe_76_ibu_tri<br><br>Show the field ONLY if:<br>[fe_76_ibup] = '1'	Ibuprofeno	checkbox<br>1 — fe_76_ibu_tri___1 — Primer trimestre<br>2 — fe_76_ibu_tri___2 — Segundo trimestre<br>3 — fe_76_ibu_tri___3 — Tercer trimestre
258	fe_76_ibu1_vez<br><br>Show the field ONLY if:<br>[fe_76_ibu_tri(1)] = '1'	Ibuprofeno: Primer trimestre<br>*vez/veces*	dropdown<br>1 — 1<br>2 — 2<br>3 — 3<br>4 — 4<br>5 — 5<br>6 — 6<br>7 — 7<br>8 — 8<br>9 — 9<br>10 — 10<br>11 — >10
259	fe_76_ibu2_vez<br><br>Show the field ONLY if:<br>[fe_76_ibu_tri(2)] = '1'	Ibuprofeno: Segundo trimestre<br>*vez/veces*	dropdown<br>1 — 1<br>2 — 2<br>3 — 3<br>4 — 4<br>5 — 5<br>6 — 6<br>7 — 7<br>8 — 8<br>9 — 9<br>10 — 10<br>11 — >10

<table>
<tr><td>260</td><td>fe_76_ibu3_vez

Show the field ONLY if:
[fe_76_ibu_tri(3)] = '1'</td><td>Ibuprofeno: Tercer trimestre
vez/veces</td><td>dropdown<table><tr><td>1</td><td>1</td></tr><tr><td>2</td><td>2</td></tr><tr><td>3</td><td>3</td></tr><tr><td>4</td><td>4</td></tr><tr><td>5</td><td>5</td></tr><tr><td>6</td><td>6</td></tr><tr><td>7</td><td>7</td></tr><tr><td>8</td><td>8</td></tr><tr><td>9</td><td>9</td></tr><tr><td>10</td><td>10</td></tr><tr><td>11</td><td>>10</td></tr></table></td></tr>
<tr><td>261</td><td>fe_76_dip_tri

Show the field ONLY if:
[fe_76_dipi] = '1'</td><td>Dipirona</td><td>checkbox<table><tr><td>1</td><td>fe_76_dip_tri___1</td><td>Primer trimestre</td></tr><tr><td>2</td><td>fe_76_dip_tri___2</td><td>Segundo trimestre</td></tr><tr><td>3</td><td>fe_76_dip_tri___3</td><td>Tercer trimestre</td></tr></table></td></tr>
<tr><td>262</td><td>fe_76_dip1_vez

Show the field ONLY if:
[fe_76_dip_tri(1)] = '1'</td><td>Dipirona: Primer trimestre
vez/veces</td><td>dropdown<table><tr><td>1</td><td>1</td></tr><tr><td>2</td><td>2</td></tr><tr><td>3</td><td>3</td></tr><tr><td>4</td><td>4</td></tr><tr><td>5</td><td>5</td></tr><tr><td>6</td><td>6</td></tr><tr><td>7</td><td>7</td></tr><tr><td>8</td><td>8</td></tr><tr><td>9</td><td>9</td></tr><tr><td>10</td><td>10</td></tr><tr><td>11</td><td>>10</td></tr></table></td></tr>
<tr><td>263</td><td>fe_76_dip2_vez

Show the field ONLY if:
[fe_76_dip_tri(2)] = '1'</td><td>Dipirona: Segundo trimestre
vez/veces</td><td>dropdown<table><tr><td>1</td><td>1</td></tr><tr><td>2</td><td>2</td></tr><tr><td>3</td><td>3</td></tr><tr><td>4</td><td>4</td></tr><tr><td>5</td><td>5</td></tr><tr><td>6</td><td>6</td></tr><tr><td>7</td><td>7</td></tr><tr><td>8</td><td>8</td></tr><tr><td>9</td><td>9</td></tr><tr><td>10</td><td>10</td></tr><tr><td>11</td><td>>10</td></tr></table></td></tr>
</table>

264	fe_76_dip3_vez<br><br>Show the field ONLY if:<br>[fe_76_dip_tri(3)] = '1'	Dipirona: Tercer trimestre<br>*vez/veces*	dropdown<br>1 | 1<br>2 | 2<br>3 | 3<br>4 | 4<br>5 | 5<br>6 | 6<br>7 | 7<br>8 | 8<br>9 | 9<br>10 | 10<br>11 | >10
265	fe_76_asp_tri<br><br>Show the field ONLY if:<br>[fe_76_aspi] = '1'	Aspirina	checkbox<br>1 | fe_76_asp_tri___1 | Primer trimestre<br>2 | fe_76_asp_tri___2 | Segundo trimestre<br>3 | fe_76_asp_tri___3 | Tercer trimestre
266	fe_76_asp1_vez<br><br>Show the field ONLY if:<br>[fe_76_asp_tri(1)] = '1'	Aspirina: Primer trimestre<br>*vez/veces*	dropdown<br>1 | 1<br>2 | 2<br>3 | 3<br>4 | 4<br>5 | 5<br>6 | 6<br>7 | 7<br>8 | 8<br>9 | 9<br>10 | 10<br>11 | >10
267	fe_76_asp2_vez<br><br>Show the field ONLY if:<br>[fe_76_asp_tri(2)] = '1'	Aspirina: Segundo trimestre<br>*vez/veces*	dropdown<br>1 | 1<br>2 | 2<br>3 | 3<br>4 | 4<br>5 | 5<br>6 | 6<br>7 | 7<br>8 | 8<br>9 | 9<br>10 | 10<br>11 | >10

268	fe_76_asp3_vez<br><br>Show the field ONLY if:<br>[fe_76_asp_tri(3)] = '1'	Aspirina: Tercer trimestre<br>*vez/veces*	dropdown<br>1 = 1<br>2 = 2<br>3 = 3<br>4 = 4<br>5 = 5<br>6 = 6<br>7 = 7<br>8 = 8<br>9 = 9<br>10 = 10<br>11 = >10
269	fe_76_dic_tri<br><br>Show the field ONLY if:<br>[fe_76_dicl] = '1'	Diclofenac	checkbox<br>1 fe_76_dic_tri___1 Primer trimestre<br>2 fe_76_dic_tri___2 Segundo trimestre<br>3 fe_76_dic_tri___3 Tercer trimestre
270	fe_76_dic1_vez<br><br>Show the field ONLY if:<br>[fe_76_dic_tri(1)] = '1'	Diclofenac: Primer trimestre<br>*vez/veces*	dropdown<br>1 = 1<br>2 = 2<br>3 = 3<br>4 = 4<br>5 = 5<br>6 = 6<br>7 = 7<br>8 = 8<br>9 = 9<br>10 = 10<br>11 = >10
271	fe_76_dic2_vez<br><br>Show the field ONLY if:<br>[fe_76_dic_tri(2)] = '1'	Diclofenac: Segundo trimestre<br>*vez/veces*	dropdown<br>1 = 1<br>2 = 2<br>3 = 3<br>4 = 4<br>5 = 5<br>6 = 6<br>7 = 7<br>8 = 8<br>9 = 9<br>10 = 10<br>11 = >10

272	fe_76_dic3_vez<br><br>Show the field ONLY if:<br>[fe_76_dic_tri(3)] = '1'	Diclofenac: Tercer trimestre<br>*vez/veces*	dropdown<br>1 — 1<br>2 — 2<br>3 — 3<br>4 — 4<br>5 — 5<br>6 — 6<br>7 — 7<br>8 — 8<br>9 — 9<br>10 — 10<br>11 — >10
273	fe_77_tit	77. ¿Vivió alguna de las siguientes situaciones traumáticas durante el embarazo de su hijo?	descriptive
274	fe_77_en	77.1 Un amigo cercano o familiar tuvo una enfermedad grave	radio<br>1 — Sí<br>2 — No<br>3 — N/c<br><br>Custom alignment: RH
275	fe_77_e1<br><br>Show the field ONLY if:<br>[fe_77_en] = '1'	Section Header: *77.1.1 Un amigo cercano o familiar tuvo una enfermedad grave*<br>Primer trimestre	radio (Matrix)<br>1 — Sí<br>2 — No
276	fe_77e2<br><br>Show the field ONLY if:<br>[fe_77_en] = '1'	Segundo trimestre	radio (Matrix)<br>1 — Sí<br>2 — No
277	fe_77_e3<br><br>Show the field ONLY if:<br>[fe_77_en] = '1'	Tercer trimestre	radio (Matrix)<br>1 — Sí<br>2 — No
278	fe_77_fa	77.2 Un amigo cercano o familiar falleció	radio<br>1 — Sí<br>2 — No<br>3 — N/c<br><br>Custom alignment: RH
279	fe_77_f1<br><br>Show the field ONLY if:<br>[fe_77_fa] = '1'	Section Header: *77.2.1 Un amigo cercano o familiar falleció*<br>Primer trimestre	radio (Matrix)<br>1 — Sí<br>2 — No
280	fe_77_f2<br><br>Show the field ONLY if:<br>[fe_77_fa] = '1'	Segundo trimestre	radio (Matrix)<br>1 — Sí<br>2 — No
281	fe_77_f3<br><br>Show the field ONLY if:<br>[fe_77_fa] = '1'	Tercer trimestre	radio (Matrix)<br>1 — Sí<br>2 — No

282	fe_77_se	77.3 Se separó de su esposo o pareja	radio<br>1 Sí<br>2 No<br>3 N/c<br><br>Custom alignment: RH
283	fe_77_s1<br><br>Show the field ONLY if:<br>[fe_77_se] = '1'	Section Header: *77.3.1 Se separó de su esposo o pareja*<br>Primer trimestre	radio (Matrix)<br>1 Sí<br>2 No
284	fe_77_s2<br><br>Show the field ONLY if:<br>[fe_77_se] = '1'	Segundo trimestre	radio (Matrix)<br>1 Sí<br>2 No
285	fe_77_s3<br><br>Show the field ONLY if:<br>[fe_77_se] = '1'	Tercer trimestre	radio (Matrix)<br>1 Sí<br>2 No
286	fe_77_tr	77.4 Su esposo o pareja perdió el trabajo	radio<br>1 Sí<br>2 No<br>3 N/c<br><br>Custom alignment: RH
287	fe_77_t1<br><br>Show the field ONLY if:<br>[fe_77_tr] = '1'	Section Header: *77.4.1 Su esposo o pareja perdió el trabajo*<br>Primer trimestre	radio (Matrix)<br>1 Sí<br>2 No
288	fe_77_t2<br><br>Show the field ONLY if:<br>[fe_77_tr] = '1'	Segundo trimestre	radio (Matrix)<br>1 Sí<br>2 No
289	fe_77_t3<br><br>Show the field ONLY if:<br>[fe_77_tr] = '1'	Tercer trimestre	radio (Matrix)<br>1 Sí<br>2 No
290	fe_77_st	77.5 Usted perdió el trabajo	radio<br>1 Sí<br>2 No<br>3 N/c<br><br>Custom alignment: RH
291	fe_77_st1<br><br>Show the field ONLY if:<br>[fe_77_st] = '1'	Section Header: *77.5.1 Usted perdió su trabajo*<br>Primer trimestre	radio (Matrix)<br>1 Sí<br>2 No
292	fe_77_st2<br><br>Show the field ONLY if:<br>[fe_77_st] = '1'	Segundo trimestre	radio (Matrix)<br>1 Sí<br>2 No

293	fe_77_st3<br><br>Show the field ONLY if:<br>[fe_77_st] = '1'	Tercer trimestre	radio (Matrix)<br>1 Sí<br>2 No
294	fe_78	78 En general, ¿cuántos amigos o familiares usted siente cercanos (a quien pueda llamar por ayuda o contención, con quienes pueda hablar)?	radio, Required<br>1 Ninguno<br>2 1-2<br>3 3-5<br>4 6-9<br>5 10 o mas<br>6 N/c
295	fe_79	79 ¿En los tres últimos meses antes del parto, ¿Cómo descansó a la noche?	radio, Required<br>1 Muy mal<br>2 Mal<br>3 Mas o menos bien<br>4 Bien<br>5 Muy bien<br>6 N/c
296	fe_80	80 En los últimos tres meses antes del parto, ¿Cuántas horas durmio a la noche?<br>*Horas*	text (number)
297	fe_80_nc	80.a N/c	checkbox<br>1 fe_80_nc___1 N/c
298	fe_81	Section Header: *Datos del padre*<br><br>81 Padre - Fecha de nacimiento<br>*DD-MM-YYYY*	text (date_dmy)
299	fe_81_nc	81.a . N/c	checkbox<br>1 fe_81_nc___1 N/c
300	fe_82	82 Padre - Educacion	radio<br>1 N/c<br>2 Analfabeto<br>3 Alfabeto sin educacion formal<br>4 Primaria completa<br>5 Primaria incompleta<br>6 Secundaria completa<br>7 Secundaria incompleta<br>8 Terciario/Universitario completo<br>9 Terciario/Universitario incompleto
301	fe_83	83 ¿Fumó usted durante el embarazo de su mujer?	radio<br>1 N/c<br>2 No<br>3 Si<br><br>Custom alignment: RH

302	fe_83_1<br><br>Show the field ONLY if:<br>[fe_83] = '3'	83.1 ¿Por cuánto tiempo fumó durante el embarazo (en meses)?	text (number)
303	fe_83_1nc<br><br>Show the field ONLY if:<br>[fe_83] = '3'	83.1.a N/c	checkbox<br>1 | fe_83_1nc___1 | N/c
304	fe_83_2<br><br>Show the field ONLY if:<br>[fe_83] = '3'	83.2 ¿ Cuántos cigarrillos por dia?	text (number)
305	fe_83_2nc<br><br>Show the field ONLY if:<br>[fe_83] = '3'	83.2.a N/c	checkbox<br>1 | fe_83_2nc___1 | N/c
306	fe_84	84 ¿Fuma actualmente?	radio<br>1 | N/c<br>2 | No<br>3 | Si<br><br>Custom alignment: RH
307	fe_84_1<br><br>Show the field ONLY if:<br>[fe_84] = '3'	84.1 ¿Cuántos cigarrillos por dia?	text (number)
308	fe_84_1nc<br><br>Show the field ONLY if:<br>[fe_84] = '3'	84.1.a N/c	checkbox<br>1 | fe_84_1nc___1 | N/c
309	fe_85	85 ¿Alguna vez su médico le diagnosticó asma?	radio<br>1 | N/c<br>2 | No<br>3 | Si<br><br>Custom alignment: RH
310	fe_86	86 ¿Usó en el último año salbutamol/ventolín en aerosol?	radio<br>1 | N/c<br>2 | No<br>3 | Si<br><br>Custom alignment: RH
311	fe_87	87 ¿Utiliza corticoides en aerosol (budesonide, fluticasona)?	radio<br>1 | N/c<br>2 | No<br>3 | Si<br><br>Custom alignment: RH
312	fe_ic	Section Header: *Llenado del formulario*<br>88 Investigador que completo	text, Required
313	fe_fc	89 Fecha de completado<br>*DD-MM-YYYY*	text (date_dmy), Required

314	epidemiologica2013_complete	Section Header: *Form Status*<br>Complete?	dropdown<br>0 Incomplete<br>1 Unverified<br>2 Complete
Instrument: **Internacion2013**			
315	in_00_hospital_numero	Hospital y Numero	text
316	in_logo		descriptive
317	in_00_iniciales	Section Header: *Ficha de internacion*<br>Iniciales	text, Required
318	in_00_dni	DNI	text, Required
319	in_01	01 Fecha de hospitalizacion<br>*DD-MM-YYYY*	text (date_dmy), Required
320	in_02	02 Fecha de nacimiento<br>*DD-MM-YYYY*	text (date_dmy), Required
321	in_03	03 Sexo	radio, Required<br>1 Femenino<br>2 Masculino<br>Custom alignment: RH
322	in_04	04 Internacion actual - Lugar	radio, Required<br>1 Sala<br>2 UCIP<br>3 UCIN<br>Custom alignment: RH
323	in_04_1	04_1 Internacion actual - Fecha<br>*DD-MM-YYYY*	text (date_dmy), Required
324	in_04_2<br>Show the field ONLY if: [in_04] = '2' or [in_04] = '3'	04_2 Internacion actual - Derivado desde	radio, Required<br>1 Sala<br>2 Guardia<br>3 Otra institucion<br>Custom alignment: RH
325	in_4_2_esp<br>Show the field ONLY if: [in_04_2] = '3'	Otra institución: Especifique	text
326	in_04_3<br>Show the field ONLY if: [in_04] = '2' or [in_04] = '3'	04_3 Si el paciente es derivado de otra sala o institución, ¿fue completada la ficha epidemiológica?	radio<br>1 No<br>2 Si<br>3 No sabe<br>Custom alignment: RH
327	in_04_4<br>Show the field ONLY if: [in_04] = '2' or [in_04] = '3'	04_4 Internacion actual - Complicacion que motiva ingreso a UCIP/UCIN:	text
328	in_05	05 Signos y síntomas al ingreso -	descriptive

329	in_05_f	05.1 Fiebre	yesno<br>1 Yes<br>0 No<br><br>Custom alignment: RH
330	in_05_fi<br><br>Show the field ONLY if:<br>[in_05_f] = '1'	05.1.1 Fiebre: especifique mayor nivel de temperatura alcanzado:<br>*°C*	text
331	in_05_tp	05.2 Taquipnea	yesno<br>1 Yes<br>0 No<br><br>Custom alignment: RH
332	in_05_tpf<br><br>Show the field ONLY if:<br>[in_05_tp] = '1'	05.2.1 Taquipnea especifique frecuencia respiratoria al ingreso (previa al tratamiento):<br>*resp/min*	text
333	in_05_tc	05.3 Taquicardia	yesno<br>1 Yes<br>0 No<br><br>Custom alignment: RH
334	in_05_tcf<br><br>Show the field ONLY if:<br>[in_05_tc] = '1'	05.3.1 Taquicardia. especifique frecuencia cardíaca al ingreso (previa al tratamiento):<br>*lat/min*	text
335	in_05_ti	05.4 Tiraje	yesno<br>1 Yes<br>0 No<br><br>Custom alignment: RH
336	in_05_tir<br><br>Show the field ONLY if:<br>[in_05_ti] = '1'	05.4.1 Tiraje	radio<br>1 Subcostal<br>2 Intercostal<br>3 Generalizado<br>4 N/c
337	in_05_si	05.5 Sibilancias	yesno<br>1 Yes<br>0 No<br><br>Custom alignment: RH
338	in_05_sib<br><br>Show the field ONLY if:<br>[in_05_si] = '1'	05.5.1 Sibilancias	radio<br>1 Espiratorias<br>2 En ambos tiempos<br>3 Audibles sin estetoscopio<br>4 N/c

339	in_05_an	05.6 Aleteo nasal	yesno<br>1 Yes<br>0 No<br><br>Custom alignment: RH
340	in_05_to	05.7 Tos	yesno<br>1 Yes<br>0 No<br><br>Custom alignment: RH
341	in_05_tos<br><br>Show the field ONLY if: [in_05_to] = '1'	05.7.1 Tos	radio<br>1 Seca<br>2 Catarral<br>3 Laríngea<br>4 Paroxística o con reprise<br>5 N/c
342	in_05_ap	05.8 Apnea	yesno<br>1 Yes<br>0 No<br><br>Custom alignment: RH
343	in_05_vo	05.9 Vómitos (no post-tusígenos)	yesno<br>1 Yes<br>0 No<br><br>Custom alignment: RH
344	in_05_di	05.10 Diarrea	yesno<br>1 Yes<br>0 No<br><br>Custom alignment: RH
345	in_05_co	05.11 Convulsiones	yesno<br>1 Yes<br>0 No<br><br>Custom alignment: RH
346	in_6	06 Fecha de comienzo de los síntomas:	text (date_dmy)
347	in_7	07 ¿Cuántas horas transcurrieron desde el inicio del cuadro hasta el día de la internacion?	radio, Required<br>1 Menos de 24<br>2 24-48<br>3 48-72<br>4 72-96<br>5 Mas de 96

348	in_8	08 ¿Cuántas veces había consultado previamente por este cuadro?	radio, Required<br>1 Ninguna<br>2 1<br>3 2<br>4 3<br>5 4 o mas
349	in_09	09 Convivientes con una infección respiratoria aguda en las 2 semanas previas	radio<br>1 N/c<br>2 No<br>3 Sí<br><br>Custom alignment: RH
350	in_09_1<br><br>Show the field ONLY if:<br>[in_09] = '3'	09.1 Detalle cuántos	text
351	in_09_2<br><br>Show the field ONLY if:<br>[in_09] = '3'	09.2 Especifique quién/es:	checkbox<br>1 in_09_2___1 Padre<br>2 in_09_2___2 Madre<br>3 in_09_2___3 Hermana/o<br>4 in_09_2___4 N/c
352	in_09_3dia<br><br>Show the field ONLY if:<br>[in_09] = '3'	09.3 Diagnóstico (dado por un médico):	text
353	in_09_nc<br><br>Show the field ONLY if:<br>[in_09] = '3'	Contestó	radio<br>1 Sí<br>2 No<br><br>Custom alignment: RH
354	in_010	10 Diagnostico Ingreso:	checkbox<br>1 in_010___1 Bronquiolitis<br>2 in_010___2 Neumonia<br>3 in_010___3 Apneas<br>4 in_010___4 Sindrome coqueluchoide<br>5 in_010___5 Otro
355	in_10_e<br><br>Show the field ONLY if:<br>[in_010(5)] = '1'	Especifique	text
356	in_11_sat	11 Saturación de oxígeno al ingreso (con FiO2 ambiental, previa al tratamiento):<br>*por ciento*	text (number, Min: 0, Max: 100)
357	in_11_ne		radio<br>1 No especificaado por ingresar en condición crítica (con O2 suplementario)<br>2 No especificado por falta de saturómetro

358	in_12_lab	Section Header: *Evolucion*<br>12 ¿Se realizó laboratorio durante la internación en esta sala?	radio, Required<br>1 N/c<br>2 No<br>3 Si<br>Custom alignment: RH
359	in_12_tr<br>Show the field ONLY if: [in_12_lab] = '3'	Transcriba los resultados del primer estudio realizado:	descriptive
360	in_12_hg<br>Show the field ONLY if: [in_12_lab] = '3'	12_1 Laboratorio - Hemoglobina	text
361	in_12_hc<br>Show the field ONLY if: [in_12_lab] = '3'	12_2 Laboratorio - Hematocrito	text
362	in_12_gb<br>Show the field ONLY if: [in_12_lab] = '3'	12_3 Laboratorio - GB_Rto<br>*Fórmula*	text
363	in_12_pm<br>Show the field ONLY if: [in_12_lab] = '3'	12_4 Laboratorio - GB_PMM<br>*%*	text
364	in_12_lin<br>Show the field ONLY if: [in_12_lab] = '3'	12_5 Laboratorio - GB_Linfocitos<br>*%*	text
365	in_12_er<br>Show the field ONLY if: [in_12_lab] = '3'	12_6 Laboratorio - Eritrosedimentacion	text
366	in_12_cr<br>Show the field ONLY if: [in_12_lab] = '3'	12_7 Laboratorio - Proteina C Reactiva	text
367	in_12_co<br>Show the field ONLY if: [in_12_lab] = '3'	12_8 CO2 arterial	text
368	in_12_cv<br>Show the field ONLY if: [in_12_lab] = '3'	12_9 CO2 venoso	text
369	in_12_in<br>Show the field ONLY if: [in_12_lab] = '3'	12_10 ¿Fue realizado al Ingreso del paciente a sala?	radio, Required<br>1 N/c<br>2 No<br>3 Si<br>Custom alignment: RH
370	in_13_rx	13 ¿Se realizó Rx de torax al ingreso?	radio, Required<br>1 N/c<br>2 No<br>3 Si<br>Custom alignment: RH

371	in_13_1<br><br>Show the field ONLY if:<br>[in_13_rx] = '3'	13_1 Especifique hallazgos Rx ingreso	checkbox<br>1 in_13_1___1 Normal<br>2 in_13_1___2 Atrapamiento aereo<br>3 in_13_1___3 Atelectasia<br>4 in_13_1___4 Infiltrado intersticial inespecifico<br>5 in_13_1___5 Infiltrado intersticial compatible con neumonitis<br>6 in_13_1___6 Imagen de condensacion compatible con neumonia<br>7 in_13_1___7 Derrame pleural<br>8 in_13_1___8 Neumotorax<br>9 in_13_1___9 Otra imagen
372	in_10_1_a<br><br>Show the field ONLY if:<br>[in_13_1(9)] = '1'	13.1.a Rx de torax - Otra imagen	text
373	in_14_o	14 Requerimiento de Oxigeno durante la internación:	radio, Required<br>1 N/c<br>2 No<br>3 Si<br><br>Custom alignment: RH
374	in_11_1<br><br>Show the field ONLY if:<br>[in_14_o] = '3'	14_1 Req. O2 - Canula	radio<br>1 Sí<br>2 No<br><br>Custom alignment: RH
375	in_11_1_a<br><br>Show the field ONLY if:<br>[in_11_1] = '1'	14_1a Req. O2 - Canula - Dias	text (number)
376	in_11_2<br><br>Show the field ONLY if:<br>[in_14_o] = '3'	14_2 Req. O2 - Mascara	radio<br>1 Sí<br>2 No<br><br>Custom alignment: RH
377	in_11_2_a<br><br>Show the field ONLY if:<br>[in_11_2] = '1'	14_2a Req. O2 - Mascara - Dias	text (number)
378	in_11_3<br><br>Show the field ONLY if:<br>[in_14_o] = '3'	14_3 Req. O2 - Mascara Venturi	radio<br>1 Sí<br>2 No<br><br>Custom alignment: RH
379	in_11_3_a<br><br>Show the field ONLY if:<br>[in_11_3] = '1'	14_3a Req. O2 - Mascara Venturi - Fi02	text (number)

380	in_11_3_b<br><br>Show the field ONLY if:<br>[in_11_3] = '1'	14_3b Req. O2 - Mascara Venturi - Dias	text (number)
381	in_11_4<br><br>Show the field ONLY if:<br>[in_14_o] = '3'	14_4 Req. O2 - Reservorio	radio<br>1 Sí<br>2 No<br><br>Custom alignment: RH
382	in_11_4_a<br><br>Show the field ONLY if:<br>[in_11_4] = '1'	14_4a Req. O2 - Reservorio - Dias	text (number)
383	in_11_5<br><br>Show the field ONLY if:<br>[in_14_o] = '3'	14_5 Req. O2 - Ventilacion No Invasiva	radio<br>1 Sí<br>2 No<br><br>Custom alignment: RH
384	in_11_5_a<br><br>Show the field ONLY if:<br>[in_11_5] = '1'	14_5a Req. O2 - Ventilacion No Invasiva - Dias	text (number)
385	in_11_6<br><br>Show the field ONLY if:<br>[in_14_o] = '3'	14_6 Req. O2 - ARM	radio<br>1 Sí<br>2 No<br><br>Custom alignment: RH
386	in_11_6_a<br><br>Show the field ONLY if:<br>[in_11_6] = '1'	14_6a Req. O2 - ARM - Dias	text (number)
387	in_11_7<br><br>Show the field ONLY if:<br>[in_14_o] = '3'	14_7 Req. O2 - Dias totales	text (number)
388	in_15_1_01f	Section Header: *Tratamientos recibidos*<br><br>15_1_01 - Antivirales - Oseltamivir<br>*Fecha inicio*	text (date_dmy)
389	in_15_1_01	15_1_01 - Antivirales - Oseltamivir<br>*Días totales*	text (number)
390	in_15_1_02f	15_1_02 - Antivirales - Otro<br>*Fecha de inicio*	text (date_dmy)
391	in_15_1_02	15_1_02 - Antivirales - Otro<br>*Días totales*	text (number)
392	in_15_2_01f	15_2_01 - Antibioticos - Penicilina<br>*Fecha de inicio*	text (date_dmy)
393	in_15_2_01	15_2_01 - Antibioticos - Penicilina<br>*Días totales*	text (number)
394	in_15_2_02f	15_2_02 - Antibioticos - Cefalotina<br>*Fecha de inicio*	text (date_dmy)
395	in_15_2_02	15_2_02 - Antibioticos - Cefalotina<br>*Días totales*	text (number)
396	in_15_2_03f	15_2_03 - Antibioticos - Ampicilina<br>*Fecha de inicio*	text (date_dmy)
397	in_15_2_03	15_2_03 - Antibioticos - Ampicilina<br>*Días totales*	text (number)

398	in_15_2_04f	15_2_04 - Antibioticos - Ampicilina - Sulbactam<br>*Fecha de inicio*	text (date_dmy)
399	in_15_2_04	15_2_04 - Antibioticos - Ampicilina - Sulbactam<br>*Días totales*	text (number)
400	in_15_2_05f	15_2_05 - Antibioticos - Amoxicilina<br>*Fecha de inicio*	text (date_dmy)
401	in_15_2_05	15_2_05 - Antibioticos - Amoxicilina<br>*Días totales*	text (number)
402	in_15_2_06f	15_2_06 - Antibioticos - Amoxicilina - Clavulanico<br>*Fecha de inicio*	text (date_dmy)
403	in_15_2_06	15_2_06 - Antibioticos - Amoxicilina - Clavulanico<br>*Días totales*	text (number)
404	in_15_2_07f	15_2_07 - Antibioticos - Ceftriaxona<br>*Fecha de inicio*	text (date_dmy)
405	in_15_2_07	15_2_07 - Antibioticos - Ceftriaxona<br>*Días totales*	text (number)
406	in_15_2_08f	15_2_08 - Antibioticos - Cefotaxime<br>*Fecha de inicio*	text (date_dmy)
407	in_15_2_08	15_2_08 - Antibioticos - Cefotaxime<br>*Días totales*	text (number)
408	in_15_2_09f	15_2_09 - Antibioticos - Clindamicina<br>*Fecha de inicio*	text (date_dmy)
409	in_15_2_09	15_2_09 - Antibioticos - Clindamicina<br>*Días totales*	text (number)
410	in_15_2_10f	15_2_10 - Antibioticos - Trimetroprima - Sulfametoxazol<br>*Fecha de inicio*	text (date_dmy)
411	in_15_2_10	15_2_10 - Antibioticos - Trimetroprima - Sulfametoxazol<br>*Días totales*	text (number)
412	in_15_2_11f	15_2_11 - Antibioticos - Gentamicina<br>*Fecha de inicio*	text (date_dmy)
413	in_15_2_11	15_2_11 - Antibioticos - Gentamicina<br>*Días totales*	text (number)
414	in_15_2_12f	15_2_12 - Antibioticos - Amikacina<br>*Fecha de inicio*	text (date_dmy)
415	in_15_2_12	15_2_12 - Antibioticos - Amikacina<br>*Días totales*	text (number)
416	in_15_2_13f	15_2_13 - Antibioticos - Vancomicina<br>*Fecha de inicio*	text (date_dmy)
417	in_15_2_13	15_2_13 - Antibioticos - Vancomicina<br>*Días totales*	text (number)
418	in_15_2_14f	15_2_14 - Antibioticos - Meropenem<br>*Fecha de inicio*	text (date_dmy)
419	in_15_2_14	15_2_14 - Antibioticos - Meropenem<br>*Días totales*	text (number)
420	in_15_2_15f	15_2_15 - Antibioticos - Claritromicina<br>*Fecha de inicio*	text (date_dmy)
421	in_15_2_15	15_2_15 - Antibioticos - Claritromicina<br>*Días totales*	text (number)
422	in_15_2_16f	15_2_16 - Antibioticos - Eritromicina<br>*Fecha de inicio*	text (date_dmy)
423	in_15_2_16	15_2_16 - Antibioticos - Eritromicina<br>*Días totales*	text (number)
424	in_15_2_17f	15_2_17 - Antibioticos - Otros<br>*Fecha de inicio*	text (date_dmy)

425	in_15_2_17	15_2_17 - Antibioticos - Otros<br>*Días totales*	text (number)
426	in_15_2_18f	15_2_18 - Antibioticos - Otros<br>*Fecha de inicio*	text (date_dmy)
427	in_15_2_19f	15_2_19 - Antibioticos - Otros<br>*Fecha de inicio*	text (date_dmy)
428	in_15_2_18	15_2_18 - Antibioticos - Otros<br>*Días totales*	text (number)
429	in_15_2_19	15_2_19 - Antibioticos - Otros<br>*Días totales*	text (number)
430	in_15_3_01f	15_3_01 - Corticoides - Inhalados - Budesonide<br>*Fecha de inicio*	text (date_dmy)
431	in_15_3_01	15_3_01 - Corticoides - Inhalados - Budesonide<br>*Días totales*	text (number)
432	in_15_3_03f	15_3_03 - Corticoides - Inhalados - Fluticasona<br>*Fecha de inicio*	text (date_dmy)
433	in_15_3_03	15_3_03 - Corticoides - Inhalados - Fluticasona<br>*Días totales*	text (number)
434	in_15_4_01f	15_4_01 - Corticoides - Sistemicos - Meprednisona<br>*Fecha de inicio*	text (date_dmy)
435	in_15_4_01	15_4_01 - Corticoides - Sistemicos - Meprednisona<br>*Días totales*	text (number)
436	in_15_4_02f	15_4_02 - Corticoides - Sistemicos - Betametasona<br>*Fecha de inicio*	text (date_dmy)
437	in_15_4_02	15_4_02 - Corticoides - Sistemicos - Betametasona<br>*Días totales*	text (number)
438	in_15_4_03f	15_4_03 - Corticoides - Sistemicos - Hidrocortisona<br>*Fecha de inicio*	text (date_dmy)
439	in_15_4_03	15_4_03 - Corticoides - Sistemicos - Hidrocortisona<br>*Días totales*	text (number)
440	in_15_5_01f	15_5_01 - Tratamiento - Otro - Salbutamol<br>*Fecha de inicio*	text (date_dmy)
441	in_15_5_01	15_5_01 - Tratamiento - Otro - Salbutamol<br>*Días totales*	text (number)
442	in_15_5_02f	15_5_02 - Tratamiento - Otro - Ipatropio<br>*Fecha de inicio*	text (date_dmy)
443	in_15_5_02	15_5_02 - Tratamiento - Otro - Ipatropio<br>*Días totales*	text (number)
444	in_15_5_03f	15_5_03 - Tratamiento - Otro - Solucion salina hipertonica<br>*Fecha de inicio*	text (date_dmy)
445	in_15_5_03	15_5_03 - Tratamiento - Otro - Solucion salina hipertonica<br>*Días totales*	text (number)
446	in_15_5_04f	15_5_04 - Tratamiento - Otro - Adrenalina nebulizada<br>*Fecha de inicio*	text (date_dmy)
447	in_15_5_04	15_5_04 - Tratamiento - Otro - Adrenalina nebulizada<br>*Días totales*	text (number)
448	in_15_5_05f	15_5_05 - Tratamiento - Otro -Hidratacion parenteral<br>*Fecha de inicio*	text (date_dmy)
449	in_15_5_05	15_5_05 - Tratamiento - Otro -Hidratacion parenteral<br>*Días totales*	text (number)

450	in_15_5_06f	15_5_06 - Tratamiento - Otro -Alimentacion por gavage o gastroclissis continua<br>*Fecha de inicio*	text (date_dmy)
451	in_15_5_06	15_5_06 - Tratamiento - Otro -Alimentacion por gavage o gastroclissis continua<br>*Días totales*	text (number)
452	in_15_5_od1	15_5 - Otros 1	text
453	in_15_5_od1f	15_5 - Otros 1<br>*Fecha de inicio*	text (date_dmy)
454	in_15_5_od1dt	15_5 - Otros 1<br>*Días totales*	text
455	in_15_5_od2	15_5 - Otros 2	text
456	in_15_5_od2f	15_5 - Otros 2<br>*Fecha de inicio*	text (date_dmy)
457	in_15_5_od2dt	15_5 - Otros 2<br>*Días totales*	text
458	in_14_ay	16 Ayuno	radio, Required<br>1 N/c<br>2 No<br>3 Si<br>Custom alignment: RH
459	in_16_a<br>Show the field ONLY if:<br>[in_14_ay] = '3'	16a Ayuno - Especifique dias totales	text (number)
460	in_17_ifi	Section Header: *Diagnostico Virologico*<br>17 Realizacion de IFI:	radio, Required<br>1 N/c<br>2 No<br>3 Si<br>Custom alignment: RH
461	in_17_1ifi<br>Show the field ONLY if:<br>[in_17_ifi] = '3'	17_1 IFI - Resultado	radio<br>1 N/c<br>2 Negativo<br>3 Positivo<br>Custom alignment: RH
462	in_17_1_ifi<br>Show the field ONLY if:<br>[in_17_1ifi] = '3'	17_1_1 Indique Virus	checkbox<br>1 in_17_1_ifi___1 Virus Sincicial Respiratorio (VSR)<br>2 in_17_1_ifi___2 Parainfluenza<br>3 in_17_1_ifi___3 Influenza A<br>4 in_17_1_ifi___4 Influenza B<br>5 in_17_1_ifi___5 Adenovirus<br>6 in_17_1_ifi___6 Rinovirus<br>7 in_17_1_ifi___7 Metapneumovirus<br>8 in_17_1_ifi___8 ordetella pertussis<br>9 in_17_1_ifi___9 otro

<table>
<tr><td>463</td><td>in_17_1_o

Show the field ONLY if:
[in_17_1_ifi(9)] = '1'</td><td>17_1_IFI Virus - Otro</td><td>text</td></tr>
<tr><td>464</td><td>in_18_h1</td><td>18 Dertección de Influenza A (H1N1) por PCR:</td><td>radio, Required<table><tr><td>1</td><td>N/c</td></tr><tr><td>2</td><td>No</td></tr><tr><td>3</td><td>Si</td></tr></table>
Custom alignment: RH</td></tr>
<tr><td>465</td><td>in_18_1_h1

Show the field ONLY if:
[in_18_h1] = '3'</td><td>18_1 H1N1 - Resultado:</td><td>radio<table><tr><td>1</td><td>N/c</td></tr><tr><td>2</td><td>Negativo</td></tr><tr><td>3</td><td>Positivo</td></tr></table>
Custom alignment: RH</td></tr>
<tr><td>466</td><td>in_19_cb</td><td>Section Header: Cultivos bacterianos
19 Cultivos - Bacterianos</td><td>radio, Required<table><tr><td>1</td><td>No</td></tr><tr><td>2</td><td>Si</td></tr></table>
Custom alignment: RH</td></tr>
<tr><td>467</td><td>in_19_1_a

Show the field ONLY if:
[in_19_cb] = '2'</td><td>19_1_a Hemocultivo - Fecha</td><td>text (date_dmy)</td></tr>
<tr><td>468</td><td>in_19_1_b

Show the field ONLY if:
[in_19_cb] = '2'</td><td>19_1_b Hemocultivos - Resultados</td><td>radio<table><tr><td>1</td><td>Positivo</td></tr><tr><td>2</td><td>Negativo</td></tr></table>
Custom alignment: RH</td></tr>
<tr><td>469</td><td>in_19_1_c

Show the field ONLY if:
[in_19_1_b] = '1'</td><td>19_1_c Hemocultivos - Germen</td><td>text</td></tr>
<tr><td>470</td><td>in_19_1pe

Show the field ONLY if:
[in_19_1_b] = '1'</td><td>1:Penicilina</td><td>radio (Matrix)<table><tr><td>1</td><td>Sensible</td></tr><tr><td>2</td><td>Resistente</td></tr></table></td></tr>
<tr><td>471</td><td>in_19_1am

Show the field ONLY if:
[in_19_1_b] = '1'</td><td>2:Ampicilina</td><td>radio (Matrix)<table><tr><td>1</td><td>Sensible</td></tr><tr><td>2</td><td>Resistente</td></tr></table></td></tr>
<tr><td>472</td><td>in_19_1as

Show the field ONLY if:
[in_19_1_b] = '1'</td><td>3:Ampicilina-Sulbactam</td><td>radio (Matrix)<table><tr><td>1</td><td>Sensible</td></tr><tr><td>2</td><td>Resistente</td></tr></table></td></tr>
<tr><td>473</td><td>in_19_1cc

Show the field ONLY if:
[in_19_1_b] = '1'</td><td>4:Ceftriaxona/Cefotaxime</td><td>radio (Matrix)<table><tr><td>1</td><td>Sensible</td></tr><tr><td>2</td><td>Resistente</td></tr></table></td></tr>
<tr><td>474</td><td>in_19_1cl

Show the field ONLY if:
[in_19_1_b] = '1'</td><td>5:Clindamicina</td><td>radio (Matrix)<table><tr><td>1</td><td>Sensible</td></tr><tr><td>2</td><td>Resistente</td></tr></table></td></tr>
</table>

475	in_19_ts<br><br>Show the field ONLY if:<br>[in_19_1_b] = '1'	6:Trimetroprima-Sulfametazol	radio (Matrix)<br>1 Sensible<br>2 Resistente
476	in_19_ge<br><br>Show the field ONLY if:<br>[in_19_1_b] = '1'	7:Gentamicina	radio (Matrix)<br>1 Sensible<br>2 Resistente
477	in_19_1ak<br><br>Show the field ONLY if:<br>[in_19_1_b] = '1'	8:Amikacina	radio (Matrix)<br>1 Sensible<br>2 Resistente
478	in_19_1va<br><br>Show the field ONLY if:<br>[in_19_1_b] = '1'	9:Vancomicina	radio (Matrix)<br>1 Sensible<br>2 Resistente
479	in_19_1me<br><br>Show the field ONLY if:<br>[in_19_1_b] = '1'	10:Meropenem	radio (Matrix)<br>1 Sensible<br>2 Resistente
480	in_19_1ce<br><br>Show the field ONLY if:<br>[in_19_1_b] = '1'	11:Cefalotina	radio (Matrix)<br>1 Sensible<br>2 Resistente
481	in_19_2_a<br><br>Show the field ONLY if:<br>[in_19_cb] = '2'	19_2_a Hemocultivos - 2 - Fecha	text (date_dmy)
482	in_19_2_b<br><br>Show the field ONLY if:<br>[in_19_cb] = '2'	19_2_b Hemocultivos - 2 - Resultado	radio<br>1 Positivo<br>2 Negativo<br><br>Custom alignment: RH
483	in_19_2_c<br><br>Show the field ONLY if:<br>[in_19_2_b] = '1'	19_2_c Hemocultivos - 2 - Germen	text
484	in_19_2pe<br><br>Show the field ONLY if:<br>[in_19_2_b] = '1'	1:Penicilina	radio (Matrix)<br>1 Sensible<br>2 Resistente
485	in_19_2am<br><br>Show the field ONLY if:<br>[in_19_2_b] = '1'	2:Ampicilina	radio (Matrix)<br>1 Sensible<br>2 Resistente
486	in_19_2as<br><br>Show the field ONLY if:<br>[in_19_2_b] = '1'	3:Ampicilina-Sulbactam	radio (Matrix)<br>1 Sensible<br>2 Resistente
487	in_19_2cc<br><br>Show the field ONLY if:<br>[in_19_2_b] = '1'	4:Ceftriaxona/Cefotaxime	radio (Matrix)<br>1 Sensible<br>2 Resistente

488	in_19_2cl<br><br>Show the field ONLY if:<br>[in_19_2_b] = '1'	5:Clindamicina	radio (Matrix)<br>1 Sensible<br>2 Resistente
489	in_19_2ts<br><br>Show the field ONLY if:<br>[in_19_2_b] = '1'	6:Trimetroprima-Sulfametazol	radio (Matrix)<br>1 Sensible<br>2 Resistente
490	in_19_2ge<br><br>Show the field ONLY if:<br>[in_19_2_b] = '1'	7:Gentamicina	radio (Matrix)<br>1 Sensible<br>2 Resistente
491	in_19_2ak<br><br>Show the field ONLY if:<br>[in_19_2_b] = '1'	8:Amikacina	radio (Matrix)<br>1 Sensible<br>2 Resistente
492	in_19_2va<br><br>Show the field ONLY if:<br>[in_19_2_b] = '1'	9:Vancomicina	radio (Matrix)<br>1 Sensible<br>2 Resistente
493	in_19_2me<br><br>Show the field ONLY if:<br>[in_19_2_b] = '1'	10:Meropenem	radio (Matrix)<br>1 Sensible<br>2 Resistente
494	in_19_2ce<br><br>Show the field ONLY if:<br>[in_19_2_b] = '1'	11:Cefalotina	radio (Matrix)<br>1 Sensible<br>2 Resistente
495	in_19_3_a<br><br>Show the field ONLY if:<br>[in_19_cb] = '2'	19_3_a Hemocultivos - 3 - Fecha	text (date_dmy)
496	in_19_3_b<br><br>Show the field ONLY if:<br>[in_19_cb] = '2'	19_3_b Hemocultivos - 3 - Resultado	radio<br>1 Positivo<br>2 Negativo<br><br>Custom alignment: RH
497	in_19_3_c<br><br>Show the field ONLY if:<br>[in_19_3_b] = '1'	19_3_c Hemocultivos - 3 - Germen	text
498	in_19_3pe<br><br>Show the field ONLY if:<br>[in_19_3_b] = '1'	1:Penicilina	radio (Matrix)<br>1 Sensible<br>2 Resistente
499	in_19_3am<br><br>Show the field ONLY if:<br>[in_19_3_b] = '1'	2:Ampicilina	radio (Matrix)<br>1 Sensible<br>2 Resistente
500	in_19_3as<br><br>Show the field ONLY if:<br>[in_19_3_b] = '1'	3:Ampicilina-Sulbactam	radio (Matrix)<br>1 Sensible<br>2 Resistente

501	in_19_3cc<br>Show the field ONLY if:<br>[in_19_3_b] = '1'	4:Ceftriaxona/Cefotaxime	radio (Matrix)<br>1 Sensible<br>2 Resistente
502	in_19_3cl<br>Show the field ONLY if:<br>[in_19_3_b] = '1'	5:Clindamicina	radio (Matrix)<br>1 Sensible<br>2 Resistente
503	in_19_3ts<br>Show the field ONLY if:<br>[in_19_3_b] = '1'	6:Trimetroprima-Sulfametazol	radio (Matrix)<br>1 Sensible<br>2 Resistente
504	in_19_3ge<br>Show the field ONLY if:<br>[in_19_3_b] = '1'	7:Gentamicina	radio (Matrix)<br>1 Sensible<br>2 Resistente
505	in_19_3ak<br>Show the field ONLY if:<br>[in_19_3_b] = '1'	8:Amikacina	radio (Matrix)<br>1 Sensible<br>2 Resistente
506	in_19_3va<br>Show the field ONLY if:<br>[in_19_3_b] = '1'	9:Vancomicina	radio (Matrix)<br>1 Sensible<br>2 Resistente
507	in_19_3me<br>Show the field ONLY if:<br>[in_19_3_b] = '1'	10:Meropenem	radio (Matrix)<br>1 Sensible<br>2 Resistente
508	in_19_3ce<br>Show the field ONLY if:<br>[in_19_3_b] = '1'	11:Cefalotina	radio (Matrix)<br>1 Sensible<br>2 Resistente
509	in_19_4_a<br>Show the field ONLY if:<br>[in_19_cb] = '2'	19_4_a Urocultivo - 4 - Fecha	text (date_dmy)
510	in_19_4_b<br>Show the field ONLY if:<br>[in_19_cb] = '2'	19_4_b Urocultivo - 4 - Resultado	radio<br>1 Positivo<br>2 Negativo<br>Custom alignment: RH
511	in_19_4_c<br>Show the field ONLY if:<br>[in_19_4_b] = '1'	19_4_c Urocultivo - 4 - Germen	text
512	in_19_4pe<br>Show the field ONLY if:<br>[in_19_4_b] = '1'	1:Penicilina	radio (Matrix)<br>1 Sensible<br>2 Resistente
513	in_19_4am<br>Show the field ONLY if:<br>[in_19_4_b] = '1'	2:Ampicilina	radio (Matrix)<br>1 Sensible<br>2 Resistente

514	in_19_4as<br><br>Show the field ONLY if:<br>[in_19_4_b] = '1'	3:Ampicilina-Sulbactam	radio (Matrix)<br>1 Sensible<br>2 Resistente
515	in_19_4cc<br><br>Show the field ONLY if:<br>[in_19_4_b] = '1'	4:Ceftriaxona/Cefotaxime	radio (Matrix)<br>1 Sensible<br>2 Resistente
516	in_19_4cl<br><br>Show the field ONLY if:<br>[in_19_4_b] = '1'	5:Clindamicina	radio (Matrix)<br>1 Sensible<br>2 Resistente
517	in_19_4ts<br><br>Show the field ONLY if:<br>[in_19_4_b] = '1'	6:Trimetroprima-Sulfametazol	radio (Matrix)<br>1 Sensible<br>2 Resistente
518	in_19_4ge<br><br>Show the field ONLY if:<br>[in_19_4_b] = '1'	7:Gentamicina	radio (Matrix)<br>1 Sensible<br>2 Resistente
519	in_19_4ak<br><br>Show the field ONLY if:<br>[in_19_4_b] = '1'	8:Amikacina	radio (Matrix)<br>1 Sensible<br>2 Resistente
520	in_19_4va<br><br>Show the field ONLY if:<br>[in_19_4_b] = '1'	9:Vancomicina	radio (Matrix)<br>1 Sensible<br>2 Resistente
521	in_19_4me<br><br>Show the field ONLY if:<br>[in_19_4_b] = '1'	10:Meropenem	radio (Matrix)<br>1 Sensible<br>2 Resistente
522	in_19_4ce<br><br>Show the field ONLY if:<br>[in_19_4_b] = '1'	11:Cefalotina	radio (Matrix)<br>1 Sensible<br>2 Resistente
523	in_19_5_a<br><br>Show the field ONLY if:<br>[in_19_cb] = '2'	19_5_a Urocultivo - 5 - Fecha	text (date_dmy)
524	in_19_5_b<br><br>Show the field ONLY if:<br>[in_19_cb] = '2'	19_5_b Urocultivo - 5 - Resultado	radio<br>1 Positivo<br>2 Negativo<br><br>Custom alignment: RH
525	in_19_5_c<br><br>Show the field ONLY if:<br>[in_19_5_b] = '1'	19_5_c Urocultivo - 5 - Germen	text
526	in_19_5pe<br><br>Show the field ONLY if:<br>[in_19_5_b] = '1'	1:Penicilina	radio (Matrix)<br>1 Sensible<br>2 Resistente

527	in_19_5am<br><br>Show the field ONLY if:<br>[in_19_5_b] = '1'	2:Ampicilina	radio (Matrix)<br>1 Sensible<br>2 Resistente
528	in_19_5as<br><br>Show the field ONLY if:<br>[in_19_5_b] = '1'	3:Ampicilina-Sulbactam	radio (Matrix)<br>1 Sensible<br>2 Resistente
529	in_19_5cc<br><br>Show the field ONLY if:<br>[in_19_5_b] = '1'	4:Ceftriaxona/Cefotaxime	radio (Matrix)<br>1 Sensible<br>2 Resistente
530	in_19_5cl<br><br>Show the field ONLY if:<br>[in_19_5_b] = '1'	5:Clindamicina	radio (Matrix)<br>1 Sensible<br>2 Resistente
531	in_19_5ts<br><br>Show the field ONLY if:<br>[in_19_5_b] = '1'	6:Trimetroprima-Sulfametazol	radio (Matrix)<br>1 Sensible<br>2 Resistente
532	in_19_5ge<br><br>Show the field ONLY if:<br>[in_19_5_b] = '1'	7:Gentamicina	radio (Matrix)<br>1 Sensible<br>2 Resistente
533	in_19_5ak<br><br>Show the field ONLY if:<br>[in_19_5_b] = '1'	8:Amikacina	radio (Matrix)<br>1 Sensible<br>2 Resistente
534	in_19_5va<br><br>Show the field ONLY if:<br>[in_19_5_b] = '1'	9:Vancomicina	radio (Matrix)<br>1 Sensible<br>2 Resistente
535	in_19_5me<br><br>Show the field ONLY if:<br>[in_19_5_b] = '1'	10:Meropenem	radio (Matrix)<br>1 Sensible<br>2 Resistente
536	in_19_5ce<br><br>Show the field ONLY if:<br>[in_19_5_b] = '1'	11:Cefalotina	radio (Matrix)<br>1 Sensible<br>2 Resistente
537	in_19_6_a<br><br>Show the field ONLY if:<br>[in_19_cb] = '2'	19_6_a LCR - 6 - Fecha	text (date_dmy)
538	in_19_6_b<br><br>Show the field ONLY if:<br>[in_19_cb] = '2'	19_6_b LCR - 6 - Resultado	radio<br>1 Positivo<br>2 Negativo<br><br>Custom alignment: RH
539	in_19_6_c<br><br>Show the field ONLY if:<br>[in_19_6_b] = '1'	19_6_c LCR - 6 - Germen	text

540	in_19_6pe<br><br>Show the field ONLY if:<br>[in_19_6_b] = '1'	1:Penicilina	radio (Matrix)<br>1 Sensible<br>2 Resistente
541	in_19_6am<br><br>Show the field ONLY if:<br>[in_19_6_b] = '1'	2:Ampicilina	radio (Matrix)<br>1 Sensible<br>2 Resistente
542	in_19_6as<br><br>Show the field ONLY if:<br>[in_19_6_b] = '1'	3:Ampicilina-Sulbactam	radio (Matrix)<br>1 Sensible<br>2 Resistente
543	in_19_6cc<br><br>Show the field ONLY if:<br>[in_19_6_b] = '1'	4:Ceftriaxona/Cefotaxime	radio (Matrix)<br>1 Sensible<br>2 Resistente
544	in_19_6cl<br><br>Show the field ONLY if:<br>[in_19_6_b] = '1'	5:Clindamicina	radio (Matrix)<br>1 Sensible<br>2 Resistente
545	in_19_6ts<br><br>Show the field ONLY if:<br>[in_19_6_b] = '1'	6:Trimetroprima-Sulfametazol	radio (Matrix)<br>1 Sensible<br>2 Resistente
546	in_19_6ge<br><br>Show the field ONLY if:<br>[in_19_6_b] = '1'	7:Gentamicina	radio (Matrix)<br>1 Sensible<br>2 Resistente
547	in_19_6ak<br><br>Show the field ONLY if:<br>[in_19_6_b] = '1'	8:Amikacina	radio (Matrix)<br>1 Sensible<br>2 Resistente
548	in_19_6va<br><br>Show the field ONLY if:<br>[in_19_6_b] = '1'	9:Vancomicina	radio (Matrix)<br>1 Sensible<br>2 Resistente
549	in_19_6me<br><br>Show the field ONLY if:<br>[in_19_6_b] = '1'	10:Meropenem	radio (Matrix)<br>1 Sensible<br>2 Resistente
550	in_19_6ce<br><br>Show the field ONLY if:<br>[in_19_6_b] = '1'	11:Cefalotina	radio (Matrix)<br>1 Sensible<br>2 Resistente
551	in_19_7_a<br><br>Show the field ONLY if:<br>[in_19_cb] = '2'	19_7_a Liq. pleural - 6 - Fecha	text (date_dmy)
552	in_19_7_b<br><br>Show the field ONLY if:<br>[in_19_cb] = '2'	19_7_b Liq. pleural - 6 - Resultado	radio<br>1 Positivo<br>2 Negativo<br><br>Custom alignment: RH

553	in_19_7_c<br><br>Show the field ONLY if:<br>[in_19_7_b] = '1'	19_7_c Liq. pleural - 6 - Germen	text
554	in_19_7pe<br><br>Show the field ONLY if:<br>[in_19_7_b] = '1'	1:Penicilina	radio (Matrix)<br>1 Sensible<br>2 Resistente
555	in_19_7am<br><br>Show the field ONLY if:<br>[in_19_7_b] = '1'	2:Ampicilina	radio (Matrix)<br>1 Sensible<br>2 Resistente
556	in_19_7as<br><br>Show the field ONLY if:<br>[in_19_7_b] = '1'	3:Ampicilina-Sulbactam	radio (Matrix)<br>1 Sensible<br>2 Resistente
557	in_19_7cc<br><br>Show the field ONLY if:<br>[in_19_7_b] = '1'	4:Ceftriaxona/Cefotaxime	radio (Matrix)<br>1 Sensible<br>2 Resistente
558	in_19_7cl<br><br>Show the field ONLY if:<br>[in_19_7_b] = '1'	5:Clindamicina	radio (Matrix)<br>1 Sensible<br>2 Resistente
559	in_19_7ts<br><br>Show the field ONLY if:<br>[in_19_7_b] = '1'	6:Trimetroprima-Sulfametazol	radio (Matrix)<br>1 Sensible<br>2 Resistente
560	in_19_7ge<br><br>Show the field ONLY if:<br>[in_19_7_b] = '1'	7:Gentamicina	radio (Matrix)<br>1 Sensible<br>2 Resistente
561	in_19_7ak<br><br>Show the field ONLY if:<br>[in_19_7_b] = '1'	8:Amikacina	radio (Matrix)<br>1 Sensible<br>2 Resistente
562	in_19_7va<br><br>Show the field ONLY if:<br>[in_19_7_b] = '1'	9:Vancomicina	radio (Matrix)<br>1 Sensible<br>2 Resistente
563	in_19_7me<br><br>Show the field ONLY if:<br>[in_19_7_b] = '1'	10:Meropenem	radio (Matrix)<br>1 Sensible<br>2 Resistente
564	in_19_7ce<br><br>Show the field ONLY if:<br>[in_19_7_b] = '1'	11:Cefalotina	radio (Matrix)<br>1 Sensible<br>2 Resistente
565	in_19_8_a<br><br>Show the field ONLY if:<br>[in_19_cb] = '2'	19_8_a Otro: - 6 - Fecha	text (date_dmy)

566	in_19_8_b<br><br>Show the field ONLY if:<br>[in_19_cb] = '2'	19_8_b Otro: - 6 - Resultado	radio<br>1 Positivo<br>2 Negativo<br><br>Custom alignment: RH
567	in_19_8_c<br><br>Show the field ONLY if:<br>[in_19_8_b] = '1'	19_8_c Otro: - 6 - Germen	text
568	in_19_8pe<br><br>Show the field ONLY if:<br>[in_19_8_b] = '1'	1:Penicilina	radio (Matrix)<br>1 Sensible<br>2 Resistente
569	in_19_8am<br><br>Show the field ONLY if:<br>[in_19_8_b] = '1'	2:Ampicilina	radio (Matrix)<br>1 Sensible<br>2 Resistente
570	in_19_8as<br><br>Show the field ONLY if:<br>[in_19_8_b] = '1'	3:Ampicilina-Sulbactam	radio (Matrix)<br>1 Sensible<br>2 Resistente
571	in_19_8cc<br><br>Show the field ONLY if:<br>[in_19_8_b] = '1'	4:Ceftriaxona/Cefotaxime	radio (Matrix)<br>1 Sensible<br>2 Resistente
572	in_19_8cl<br><br>Show the field ONLY if:<br>[in_19_8_b] = '1'	5:Clindamicina	radio (Matrix)<br>1 Sensible<br>2 Resistente
573	in_19_8ts<br><br>Show the field ONLY if:<br>[in_19_8_b] = '1'	6:Trimetroprima-Sulfametazol	radio (Matrix)<br>1 Sensible<br>2 Resistente
574	in_19_8ge<br><br>Show the field ONLY if:<br>[in_19_8_b] = '1'	7:Gentamicina	radio (Matrix)<br>1 Sensible<br>2 Resistente
575	in_19_8ak<br><br>Show the field ONLY if:<br>[in_19_8_b] = '1'	8:Amikacina	radio (Matrix)<br>1 Sensible<br>2 Resistente
576	in_19_8va<br><br>Show the field ONLY if:<br>[in_19_8_b] = '1'	9:Vancomicina	radio (Matrix)<br>1 Sensible<br>2 Resistente
577	in_19_8me<br><br>Show the field ONLY if:<br>[in_19_8_b] = '1'	10:Meropenem	radio (Matrix)<br>1 Sensible<br>2 Resistente
578	in_19_8ce<br><br>Show the field ONLY if:<br>[in_19_8_b] = '1'	11:Cefalotina	radio (Matrix)<br>1 Sensible<br>2 Resistente

<table>
<tr><td>579</td><td>in_18</td><td>19 Complicaciones</td><td>radio, Required<table><tr><td>1</td><td>N/c</td></tr><tr><td>2</td><td>No</td></tr><tr><td>3</td><td>Si</td></tr></table>Custom alignment: RH</td></tr>
<tr><td>580</td><td>in_19_1_co

Show the field ONLY if:
[in_18] = '3'</td><td>19_1 Complicaciones :</td><td>checkbox<table><tr><td>1</td><td>in_19_1_co___1</td><td>Neumonia (si no fue el diagnóstico de ingreso)</td></tr><tr><td>2</td><td>in_19_1_co___2</td><td>Derrame pleural</td></tr><tr><td>3</td><td>in_19_1_co___3</td><td>Empiema</td></tr><tr><td>4</td><td>in_19_1_co___4</td><td>Neumotórax</td></tr><tr><td>5</td><td>in_19_1_co___5</td><td>Absceso pulmonar</td></tr><tr><td>6</td><td>in_19_1_co___6</td><td>Sepsis severa/ Shock séptico</td></tr><tr><td>7</td><td>in_19_1_co___7</td><td>Convulsiones</td></tr><tr><td>8</td><td>in_19_1_co___8</td><td>Encefalitis</td></tr><tr><td>9</td><td>in_19_1_co___9</td><td>Hipertensión Pulmonar</td></tr><tr><td>10</td><td>in_19_1_co___10</td><td>Otras:</td></tr></table></td></tr>
<tr><td>581</td><td>in_18_8a

Show the field ONLY if:
[in_19_1_co(10)] = '1'</td><td>18_1 Complicaciones - Otras - Especifique</td><td>text</td></tr>
<tr><td>582</td><td>in_20_de</td><td>20 Derivacion</td><td>radio, Required<table><tr><td>2</td><td>No</td></tr><tr><td>3</td><td>Si</td></tr></table>Custom alignment: RH</td></tr>
<tr><td>583</td><td>in_20_fe

Show the field ONLY if:
[in_20_de] = '3'</td><td>20_1 Fecha de derivación:
DD-MM-YYYY</td><td>text (date_dmy)</td></tr>
<tr><td>584</td><td>in_20_sa

Show the field ONLY if:
[in_20_de] = '3'</td><td>20_2 Sala de derivacion:</td><td>radio<table><tr><td>1</td><td>UCIP</td></tr><tr><td>2</td><td>UCIN</td></tr><tr><td>3</td><td>PEDIATRIA</td></tr></table>Custom alignment: RH</td></tr>
<tr><td>585</td><td>in_20_ho

Show the field ONLY if:
[in_20_de] = '3'</td><td>20_3 Hospiital de derivacion</td><td>text</td></tr>
<tr><td>586</td><td>in_20_ca

Show the field ONLY if:
[in_20_de] = '3'</td><td>20_4 Causa de derivacion</td><td>text</td></tr>
</table>

587	in_21_mu	21 Muerte del paciente	radio, Required<br>1 No<br>2 Si<br><br>Custom alignment: RH
588	in_21_fe<br><br>Show the field ONLY if:<br>[in_21_mu] = '2'	21_1 Fecha de deceso<br>*DD-MM-YYYY*	text (date_dmy)
589	in_21_ca<br><br>Show the field ONLY if:<br>[in_21_mu] = '2'	21_2 Causa de Muerte	text
590	in_22_fe	22 Fecha de Alta<br>*DD-MM-YYYY*	text (date_dmy), Required
591	in_23	23 Dias totales de Internacion en Sala de Pediatría:	text
592	in_24	24 Dias totales de IInternacion - UCIP - UCIN	text
593	in_25	25 Dias - Internacion - terapia - Intermedia	text
594	in_26	Section Header: *Condiciones de alta (para llenar sólo al momento del alta hospitalaria):*<br><br>26 Saturacion - Oxigeno al alta (FiO2 ambiental)	text, Required
595	in_27	27 Requerimiento de Oxigeno domiciliario	radio, Required<br>1 No<br>2 Si<br><br>Custom alignment: RH
596	in_28_a	Section Header: *28 Resultado de la PCR*<br><br>a. VSR	radio (Matrix)<br>1 SI<br>2 NO
597	in_28_b	b. H3N2 estacional	radio (Matrix)<br>1 SI<br>2 NO
598	in_28_c	c. H1 N1 estacional	radio (Matrix)<br>1 SI<br>2 NO
599	in_28_d	d. H1 N1 pandémico	radio (Matrix)<br>1 SI<br>2 NO
600	in_28_e	e. FLU B	radio (Matrix)<br>1 SI<br>2 NO
601	in_28_f	f. Metapneumovirus	radio (Matrix)<br>1 SI<br>2 NO
602	in_28_g	g. Rhinovirus	radio (Matrix)<br>1 SI<br>2 NO

603	in_28_h	h. Parainfluenza 1	radio (Matrix)<br>1 SI<br>2 NO
604	in_28_i	i. Parainfluenza 2	radio (Matrix)<br>1 SI<br>2 NO
605	in_28_j	j. Parainfluenza 3	radio (Matrix)<br>1 SI<br>2 NO
606	in_28_k	k. Bordetella pertussis	radio (Matrix)<br>1 SI<br>2 NO
607	in_ic	Investigador que completo	text, Required
608	in_fc	Fecha de completado<br>*DD-MM-YYYY*	text (date_dmy), Required
609	internacion2013_complete	Section Header: *Form Status*<br>Complete?	dropdown<br>0 Incomplete<br>1 Unverified<br>2 Complete
